# Association between air pollution and incident cardiovascular diseases among a population with cardiovascular-kidney-metabolic syndrome stages 0–3: the first evidence from the China Health and Retirement Longitudinal Study

**DOI:** 10.3389/fendo.2026.1852623

**Published:** 2026-06-23

**Authors:** Hongbo Huang, Yunhai Li, Ze Zhang, Yijing Xu, Linfeng Xie, Ying Huang, Tingting Wei, Haonan Pan, Zhiqi Hu, Zhen Gong, Jiaying Li, Yichen Wang, Aijie Zhang, Fan Li

**Affiliations:** 1 Department of Breast and Thyroid Surgery, The First Affiliated Hospital of Chongqing Medical University, Chongqing, China; 2 Department of Cardiology, The First Affiliated Hospital of Chongqing Medical University, Chongqing, China; 3 Cancer Center, Union Hospital, Tongji Medical College, Huazhong University of Science and Technology, Wuhan, China; 4 College of Public Health, Guangzhou Medical University, Guangzhou, China; 5 Department of Health Management Center, University-Town Hospital Affiliated to Chongqing Medical University, Chongqing, China

**Keywords:** air pollution, cardiovascular diseases, cardiovascular–kidney–metabolic, CHARLS, long-term exposure

## Abstract

**Background:**

The longitudinal associations between air pollution exposure and incident cardiovascular diseases (CVDs) among individuals with cardiovascular–kidney–metabolic (CKM) syndrome remains limited. This study aimed to estimate the associations of long-term exposure to air pollution with the incidence of total CVDs, heart disease, and stroke among Chinese individuals with CKM syndrome stages 0–3.

**Methods:**

This population-based cohort study used data from the China Health and Retirement Longitudinal Study (CHARLS, 2011–2018), matching air pollutant data from the China High Air Pollutants dataset. We applied single**–** and multiple**–**pollutant Cox proportional hazards model with time-varying to investigate whether annual mean exposure to PM_1_, PM_2.5_, PM_10_, O_3_, and NO_2_ affects total CVDs and its major subtypes. Subgroup and mediation analyses were also performed to further evaluate the interactions among these factors.

**Results:**

Among 7400 adults with CKM syndrome stages 0–3, 1611 (21.8%) reported CVD events (1212 heart disease and 551 stroke) during a 7-years follow-up survey. Per 10-μg/m^3^ increase in PM_1_, PM_2.5_, PM_10_, O_3_, and NO_2_ corresponded to a 14.8% (hazard ratio [HR], 1.148 [95% CI, 1.086–1.214]), 9.6% (HR, 1.096 [95% CI, 1.064–1.128]), 6.8% (HR, 1.068 [95% CI, 1.052–1.084]), 2.5% (HR, 1.025 [95% CI, 0.962–1.091]), and 12.4% (HR, 1.124 [95% CI 1.063–1.189]) higher incident risk of CVD, respectively, whereas O_3_ showed no significant association. We detected significant effect modification by education in the associations of incident CVD and heart disease with PM_1_, PM_2.5_, PM_10_, and NO_2_ (P interaction <0.05), suggesting that those with lower education level were more vulnerable. Metabolic syndrome partially mediated the association between air pollution exposure and CVD, highlighting its significance in CVDs risk assessment.

**Conclusions:**

In this nationwide cohort, long-term exposure to PM_1_, PM_2.5_, PM_10_, and NO_2_ was associated with a higher risk of incident CVD among middle-aged and older Chinese adults with CKM syndrome stages 0–3, which was partially mediated by metabolic syndrome. These findings highlight the potential vulnerability of individuals with lower educational attainment within preclinical CKM syndrome population and underscore the contribution of metabolic syndrome to pollution-related CVD risk.

## Graphical Abstract

**Figure:**
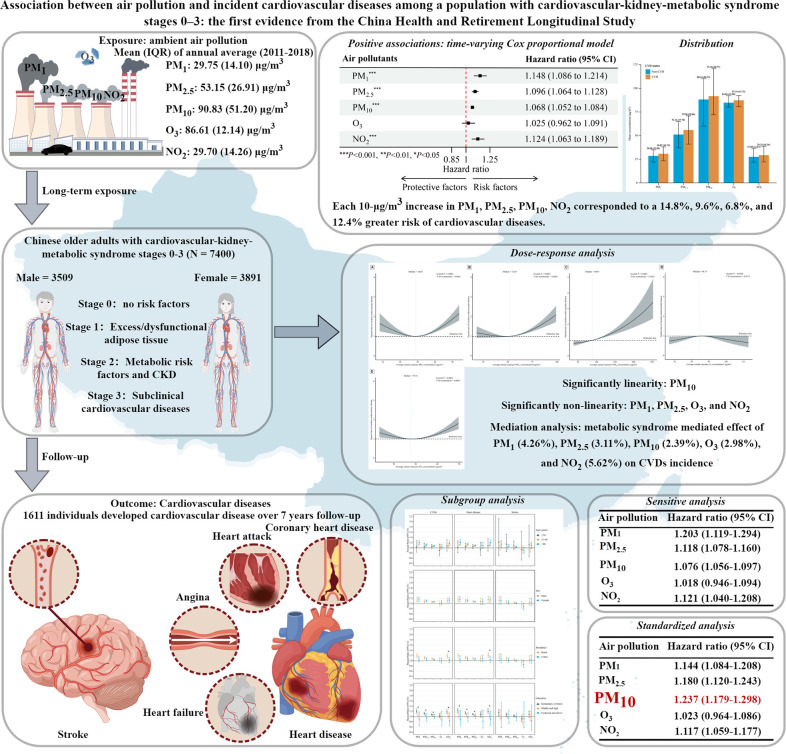

## 1 Introduction

Cardiovascular diseases (CVDs), principally ischemic heart disease and stroke, have remained the predominant cause of death and disability**-**adjusted life-years (DALYs) worldwide for decades (1–4), accounting for an estimated 19.2 million deaths and 437 million DALYs in 2023 (5). While traditional cardiovascular risk factors such as unhealthy lifestyle, high body mass index (BMI), hypertension, diabetes, and dyslipidemia have been well-established as risk factors for CVD (1, 3, 6), there is growing recognition of the critical implications of potential environmental determinants (5–14). Notably, ambient and household air pollution constitutes a major environmental contributor to CVD, resulting in approximately 3.98 million (25.2%) CVD**–**related deaths and 90.5 million (26.1%) CVD**–**related DALYs globally in 2023 (5).

In 2023, the American Heart Association (AHA) proposed the cardiovascular–kidney–metabolic (CKM) syndrome framework, which stages individuals from 0 (no risk factors) to 4 (established clinical CVD) and systematically describes the interrelated pathophysiology of metabolic disorders, chronic kidney disease, and cardiovascular dysfunction (15). Specifically, individuals across CKM syndrome stages exhibit substantially elevated lifetime risks for CVD and multiorgan complications; consequently, the AHA emphasized that preclinical risk prediction and recommends that research in CKM stages 0–3 prioritize prevention of CVD-associated events (15, 16). The CKM syndrome represent a major public health challenge, with nearly 90% of middle-aged and older adults in China affected by some stage of CKM (17). This observation underscores the importance of modifiable risk factor management in preventing the progression of CKM and occurrence of CVDs. Although numerous studies have examined air pollution components in separate relation to single CKM syndrome related conditions—such as chronic kidney disease (9), metabolic syndrome (18), diabetes (19), and hypertension (13, 20); comprehensive assessments that consider CKM syndrome as an integrated and staging construct remain limited (6). To the best of our knowledge, few population-based investigations have quantified the cumulative burden of long-term air pollution on incident CVD specifically among individuals within the CKM syndrome stages 0–3. Furthermore, it was estimated that more than 3.7 million premature mortality due to ambient air pollution occurred annually among those living in low- and middle-income countries (e.g., the greatest numbers in Southeast Asia and Western Pacific regions, including China), which experienced higher vulnerability to long-term ambient air pollution (21). However, the recent global mortality estimations attributed to ambient air pollution might be substantially biased if applying relative risks derived exclusively from epidemiological evidence conducted in developed countries (7, 11).

Although the persistence of adverse influence of ambient air pollution exposure on incident CVD has been well-documented (7–14, 20, 22), no previous studies have focused on over 875 million Chinese populations with CKM syndrome stages 0–3 (23). The objective of the present study was to evaluate the evaluate the longitudinal associations between annual concentrations of particles with aerodynamic diameters ≤1.0 μm (PM_1_), ≤2.5 μm (PM_2.5_), and ≤10 μm (PM_10_), as well as ozone (O_3_) and nitrogen dioxide (NO_2_), and the incidence of CVD through both single- and multi-pollutant models among a large, nationally representative sample of middle-aged and older Chinese adults with CKM stages 0–3. We also compared the relative importance of these exposures and examined potential effect modification by sociodemographic and individual risk factors to identify subgroups who may benefit most from targeted health policymaking and interventions.

## 2 Method

### 2.1 Study population and design

**Figure 1:**
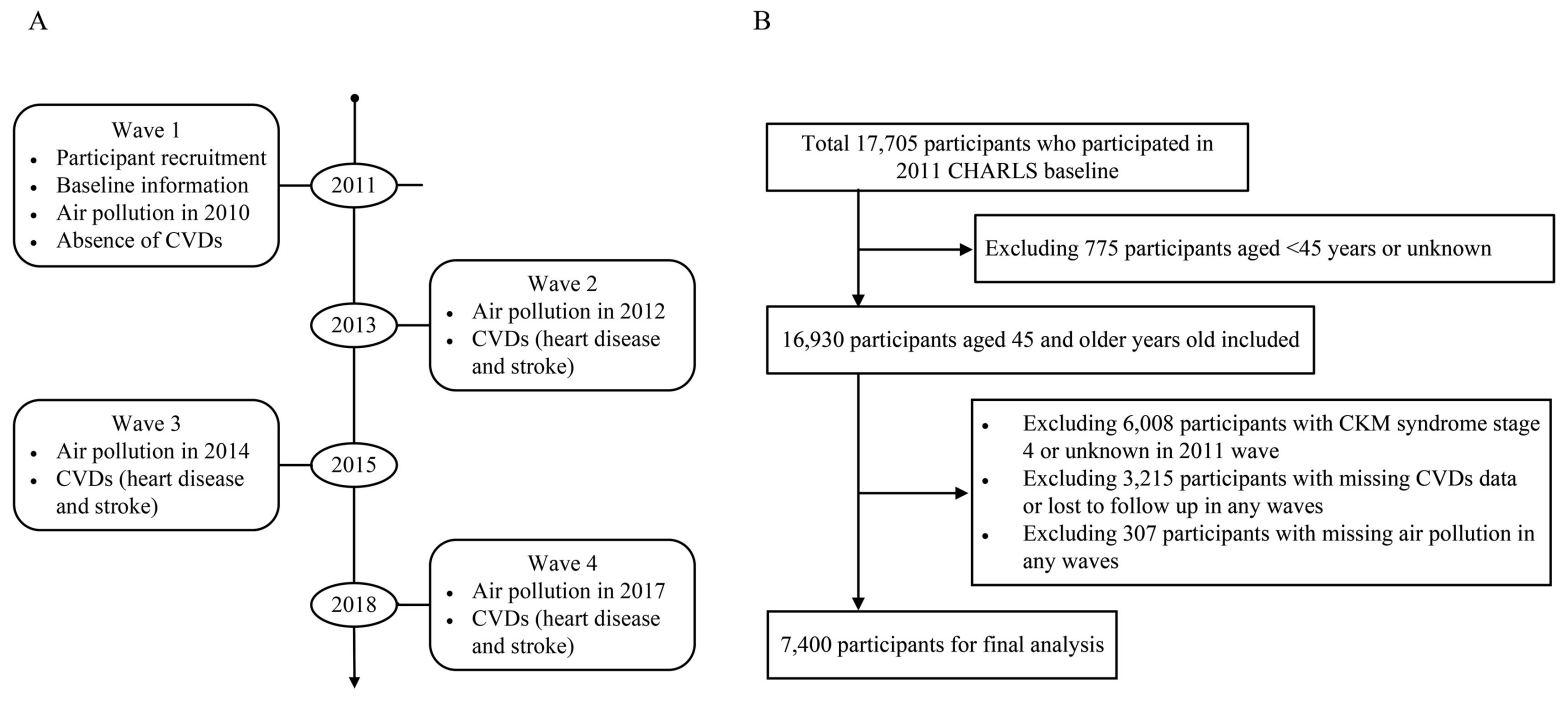
Study profile: timeline **(A)** and flowchart **(B)** of this study. CHARLS, China Health and Retirement Longitudinal Survey; CKM, cardiovascular-kidney-metabolic syndrome; CVD, cardiovascular disease.

The data used in this study were recruited from the China Health and Retirement Longitudinal Survey (CHARLS), a program carried out by the National Development Institute at Peking University (https://charls.pku.edu.cn/) (24). In summary, the program aims to assess the demographic characteristics, socioeconomic condition, health status of Chinese adults aged ≥45 years using multistage, stratified, and clustered sampling across 150 counties/districts in 28 provinces. The baseline wave (2011) was followed by computer-assisted personal interviews in 2013 (wave 2), 2015 (wave 3), and 2018 (wave 4). The study protocol was approved by the Biomedical Ethics Review Committee of Peking University (IRB00001052–11015), and written informed consent was obtained from all participants. In this study, all participants involved were tracked using unique identifiers through four waves, starting with 17,705 individuals in 2011 (**Figure 1**). However, we excluded those at CKM syndrome stage 4 (clinical CVD) at baseline or with missing CKM syndrome stage information (n=6008), those <45 years old (n=775), those lacking of air pollutant data (n=307), and/or with missing CVD information (n=3215); yielding 7400 participants for the final analysis. Partially missing covariates were imputed using the Last Observation Carried Forward method (25, 26). Age and sex were assigned from any available wave; other covariates were imputed from 2011 baseline values, given the most conditions were relative stable or exhibited minimal change (27). For each participant, we categorized the CKM syndrome stage followed AHA guidance (15, 28): stage 0 (normal BMI and waist circumference absence of any risk factors such as hypertension or diabetes), stage 1 (excess or dysfunctional adiposity), stage 2 (metabolic risk factors and/or moderate- to high-risk chronic kidney disease), stage 3 (subclinical CVD, classified as very high-risk chronic kidney disease or high predicted 10-year CVD risk), and stage 4 (established CVD, e.g., heart disease and stroke). The detailed descriptions of CKM syndrome stage 0–3 definitions, adapted to data available in CHARLS, are shown in the **Supplementary Table 1**. A range of important covariates were selected based on previous evidence (7–9, 11, 14, 27, 29, 30), including socio-demographic characteristics (age group, sex, residence, marital status, and education status), insurance, income group, cooking fuel, employment status, sleep duration, smoking status, and alcohol consumption.

### 2.2 Exposure to ambient air pollutants

We examined five common air pollutant components, namely PM_1_, PM_2.5_, PM_10_, O_3_ and NO_2_, which was associated with the development of metabolic syndrome (18), frailty (27), Parkinson’s disease (31), cardiovascular (7, 8, 10, 11), respiratory (32), and inflammatory processes (33). For each participant, we assigned high-resolution annual averages for ground-level PM_1_, PM_2.5_, PM_10_, and O_3_ at 1-km scale (0.01° × 0.01°), and ground-level NO_2_ at 10-km scale (0.1° × 0.1°), available for China from 2010 through 2018 (34), were acquired from the China High Air Pollutants (CHAP) dataset (https://weijing-rs.github.io/product.html). The CHAP dataset has been acknowledged as a robust and reliable source of air pollution data for China, and it has been widely applied to estimate the national disease burden attributable to air pollution exposure in several related studies (27, 35, 36). The10-fold cross-validation R^2^ and Root-Mean-Square Error metrics for each pollutant are summarized in **Supplementary Table 2**. Considered the lag effect of air pollutant exposure, participants were assigned the annual average pollutant concentration from the previous year (e.g., 2011 wave matched to the 2010 air pollution data) based on geocoded community-level residence information from the CHARLS PSU dataset (27, 29, 35, 37). The city-level distribution maps of annual pollutant concentrations in China across different years applied in this study are presented in **Supplementary Figure 1**.

### 2.3 Main measurement outcomes

The primary endpoint was the incident CVD (heart disease or stroke) during the follow-up period. In the CHARLS cohort, information regarding the historical physician diagnosis of CVD was ascertained via standardized questionnaires: “Have you been diagnosed with [heart attack, coronary heart disease, angina, congestive heart failure, other heart problems or stroke] by a doctor?” (36, 38, 39).

### 2.4 Statistical analysis

Descriptive analyses were performed for baseline characteristics. The pairwise correlation among the five air pollutants were evaluated using the Spearman coefficients. We employed the time-dependent Cox proportional hazards regression models for each pollutant separately to calculate the association with the incident total CVD, heart disease, and stroke among the CKM syndrome stages 0–3 individuals (11, 14, 31, 40, 41), with the survey waves serving as the timescale. We estimated the associations using four models: Model I was crude model without adjustment. Model II was adjusted for age groups (≤50, 51–60, >60) and sex (male and female). Model III was additionally adjusted for (elementary or below; middle–high school; technical school or above), residence (urban or rural), marital status (unmarried or married), insurance (no or yes), income (≤2700, 2700–4700, 4700–8000, >8000), cooking fuel (clean, solid or other), employment (unemployed or employed), and sleep duration (<7 or ≥7 hours). Model IV was additionally including smoking status (ever or never smoking) and alcohol consumption (ever or never drinking). The hazard ratios (HRs) and corresponding 95% confidence intervals (CIs) were reported for the CVD outcome associated with per 10-µg/m^3^ increase in ambient air pollutant exposure. Finally, we additionally standardized annual concentrations to compare the relative magnitude of associations across pollutants and identify the components most strongly associated with CVD risk (18).

Subgroup and interaction analyses assessed effect modification by age group, sex, residence, and education for total CVD and its subtypes. Potential nonlinearity was evaluated using restricted cubic splines (three knots at the 10th, 50th, and 90th percentiles) with likelihood-ratio tests. Subsequently, multiple pollutant models (including bi-pollutant and tri-pollutant models) were constructed with fully adjustment (Model IV) to address collinearity among pollutants (11, 18, 27). In sensitivity analyses, we excluded participants who developed CVD during the first 2 follow-up waves (n=438) and extended 2-year lag effects to assess robustness of the main results (11, 27). We also performed a mediation analysis to ascertain the potential role of metabolic syndrome as a mediator in the association between air pollution and CVD risk.

All analyses were performed using the statistical packages R version 4.3.3 alongside Stata 18.0 (Stata Corporation, College Station, Texas, USA), and a two-tailed P < 0.05 was determined to indicate statistically significant.

## 3 Results

### 3.1 Study population and exposure

We included 7400 participants with CKM syndrome stage 0–3, mean (SD) age at the baseline was 58.3 (8.8] years and 47.4% were male (**Table 1**). We discovered that higher lifetime probability of CVD was observed among women, older adults, rural and midland residents, unmarried individuals, those with elementary-level education or less, the unemployed, non-drinkers, and those sleeping <7 hours (**Table 1**). In 2011, median (inter-quartile range) exposures were 30.31 (14.24) μg/m^3^ for PM_1_, 54.91 (26.72) μg/m^3^ for PM_2.5_, 94.60 (48.20) μg/m^3^ for PM_10_, 83.19 (8.51) μg/m^3^ for O_3_, and 28.22 (15.34) μg/m^3^ for NO_2_ (**Table 1**). Over 7 years follow-up, 1,611 participants (21.8%) reported incident CVD, corresponding to 184 events per 1,000 person-years. Pollutants were moderately to highly correlated (Spearman correlation coefficient 0.524–0.973; **Supplementary Table 3**). Average annual PM_2.5_, PM_10_, O_3_, and NO_2_ exceeded WHO air quality guideline (AQG) levels for all participants; O_3_ also exceeded WHO interim targets in 84.8% (**Supplementary Table 4**). Participants with adverse events experienced higher long-term exposures across pollutants (**Supplementary Figure 2**).

**Table 1:** Baseline characteristic stratified by the incidence of cardiovascular diseases.

Characteristics	Participants, no. (%)			P value
	Overall (n = 7400)	Non-CVD (n = 5789)	CVD (n = 1611)	
Age (mean (SD))	58.3 (8.8)	57.8 (8.8)	60.0 (8.6)	<0.001
Age group, y				
≤50	1663 (22.5)	1410 (24.4)	253 (15.7)	<0.001
51 ~ 60	3024 (40.9)	2397 (41.4)	627 (38.9)	
>60	2713 (36.7)	1982 (34.2)	731 (45.4)	
BMI (mean (SD))	24.2 (36.4)	23.8 (23.8)	25.8 (64.5)	0.056
Sex (%)				
Male	3509 (47.4)	2820 (48.7)	689 (42.8)	<0.001
Female	3891 (52.6)	2969 (51.3)	922 (57.2)	
Residence				
Rural	4937 (66.7)	3883 (67.1)	1054 (65.4)	0.225
Urban	2463 (33.3)	1906 (32.9)	557 (34.6)	
Marital status				
Unmarried	801 (10.8)	591 (10.2)	210 (13.0)	0.001
Married	6599 (89.2)	5198 (89.8)	1401 (87.0)	
Education status				
Elementary school or below	5135 (69.4)	4006 (69.2)	1129 (70.1)	0.007
Middle school and high school	2044 (27.6)	1627 (28.1)	417 (25.9)	
Technical school and above	221 (3.0)	156 (2.7)	65 (4.0)	
Regional				
East	2572 (34.8)	2058 (35.6)	514 (31.9)	0.001
Midland	2665 (36.0)	2024 (35.0)	641 (39.8)	
West	2163 (29.2)	1707 (29.5)	456 (28.3)	
Cooking fuel				
Clean fuel	3099 (41.9)	2455 (42.4)	644 (40.0)	0.064
Solid fuel	4239 (57.3)	3292 (56.9)	947 (58.8)	
Other fuel	46 (0.6)	30 (0.5)	16 (1.0)	
Income group				
≤2700	1561 (21.1)	1250 (21.6)	311 (19.3)	0.396
2700 ~ 4700	1646 (22.2)	1276 (22.0)	370 (23.0)	
4700 ~ 8000	1542 (20.8)	1199 (20.7)	343 (21.3)	
>8000	1586 (21.4)	1237 (21.4)	349 (21.7)	
Employment status				
Unemployed	2156 (29.1)	1570 (27.1)	586 (36.4)	<0.001
Employed	5144 (69.5)	4151 (71.7)	993 (61.6)	
Insurance				
No	410 (5.5)	320 (5.5)	90 (5.6)	0.676
Yes	6966 (94.1)	5452 (94.2)	1514 (94.0)	
Smoking				
Ever smoking	2882 (38.9)	2286 (39.5)	596 (37.0)	0.074
Never smoking	4518 (61.1)	3503 (60.5)	1015 (63.0)	
Drinking status				
Ever drinking	2926 (39.5)	2332 (40.3)	594 (36.9)	0.046
Never drinking	4470 (60.4)	3454 (59.7)	1016 (63.1)	
Sleep duration				
<7	3564 (48.2)	2734 (47.2)	830 (51.5)	<0.001
≥7	3601 (48.7)	2891 (49.9)	710 (44.1)	
C reactive protein (mean (SD))	2.6 (7.1)	2.5 (7.0)	2.9 (7.3)	0.076
Waist circumference (mean (SD))	84.3 (12.3)	83.7 (12.1)	86.5 (12.8)	<0.001
HDL (mean (SD))	51.1 (15.4)	51.4 (15.4)	50.2 (15.2)	0.013
Fasting blood glucose (mean (SD))	110.1 (36.0)	109.3 (34.4)	112.9 (41.0)	0.001
SBP (mean (SD))	130.0 (20.8)	128.9 (20.4)	133.9 (21.9)	<0.001
DBP (mean (SD))	75.8 (12.1)	75.4 (11.9)	77.3 (12.6)	<0.001
Hypertension				
No	2799 (37.8)	2349 (40.6)	450 (27.9)	<0.001
Yes	4333 (58.6)	3237 (55.9)	1096 (68.0)	
Diabetes				
No	5161 (69.7)	4099 (70.8)	1062 (65.9)	<0.001
Yes	1146 (15.5)	854 (14.8)	292 (18.1)	
Metabolism				
No	5130 (69.3)	4103 (70.9)	1027 (63.7)	<0.001
Yes	2270 (30.7)	1686 (29.1)	584 (36.3)	
eGFR (mean (SD))	97.0 (13.6)	97.4 (13.5)	95.6 (13.6)	<0.001
Kidney disease				
No	5843 (79.0)	4624 (79.9)	1219 (75.7)	0.001
Yes	452 (6.1)	337 (5.8)	115 (7.1)	
Heart disease				
No	6188 (83.6)	5789 (100.0)	399 (24.8)	<0.001
Yes	1212 (16.4)	0 (0)	1212 (75.2)	
Stroke				
No	6849 (92.6)	5789 (100.0)	1060 (65.8)	<0.001
Yes	551 (7.4)	0 (0)	551 (34.2)	
CKM stage				
0	540 (7.3)	475 (8.2)	65 (4.0)	<0.001
1	1099 (14.9)	912 (15.8)	187 (11.6)	
2	5286 (71.4)	4052 (70.0)	1234 (76.6)	
3	475 (6.4)	350 (6.0)	125 (7.8)	
Air pollution (μg/m^3^)	Median (interquartile range)			
PM_1_	30.31 (14.24)	30.27 (13.93)	31.15 (14.84)	<0.001
PM_2.5_	54.91 (26.72)	53.25 (26.61)	58.92 (29.67)	<0.001
PM_10_	94.60 (48.20)	93.14 (47.82)	99.61 (47.13)	<0.001
O_3_	83.19 (8.51)	83.05 (8.51)	83.33 (8.13)	0.24
NO_2_	28.22 (15.34)	27.57 (15.07)	28.93 (17.00)	<0.001

The data of air pollutants presents the median (interquartile range) of annual average concentration for the baseline 2011 wave. Abbreviations: CVD, cardiovascular disease; BMI, body measure index; PM_1_, particle with aerodynamic diameter ≤1.0 μm; PM_2.5_, particle with aerodynamic diameter ≤2.5 μm; PM_10_, particle with aerodynamic diameter ≤10 μm; O_3_, ozone, and NO_2_, nitrogen dioxide.

### 3.2 Single-pollutant model of associations between air pollution and incident CVD

**Figure 2:**
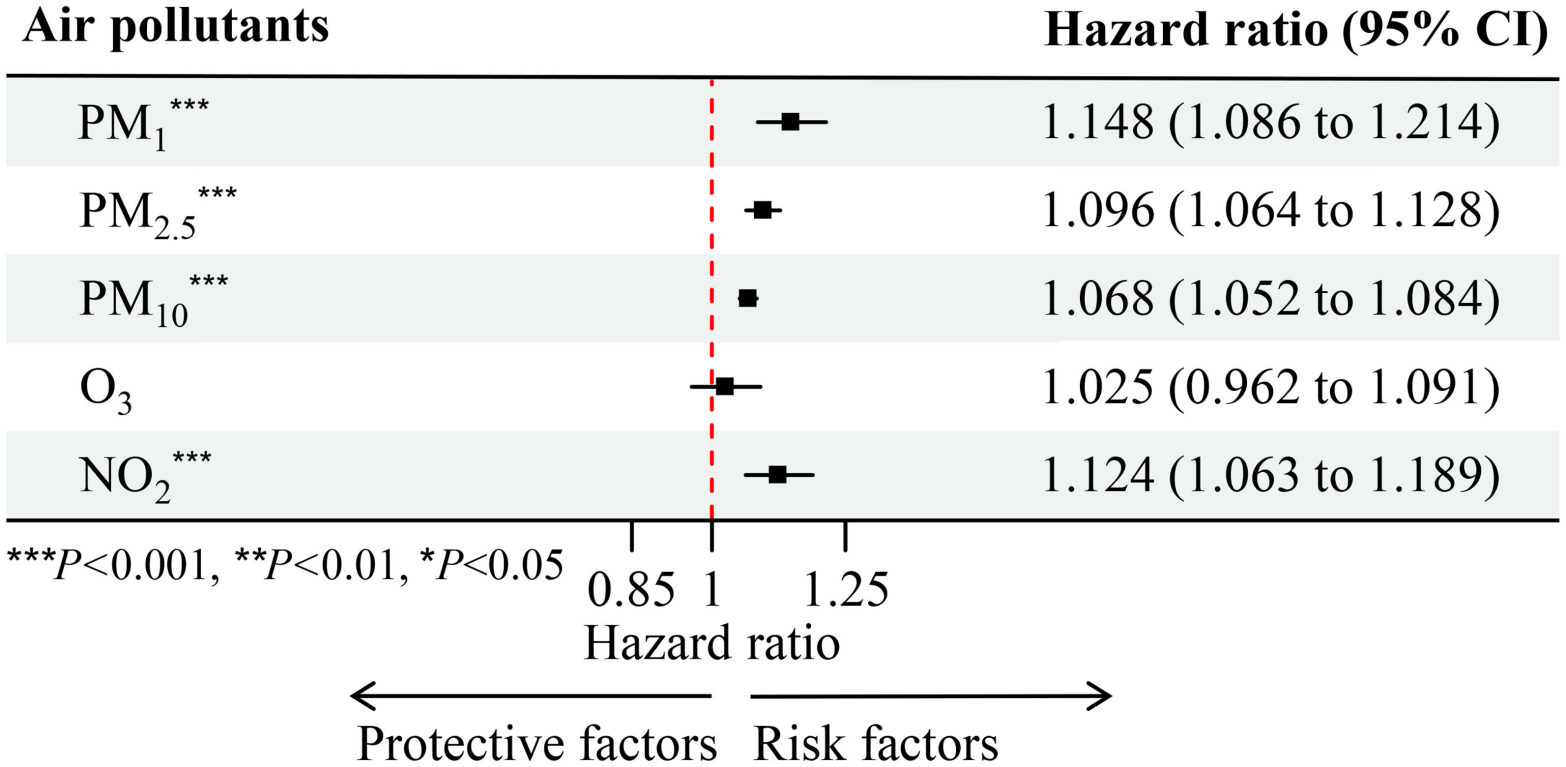
The associations between air pollutant (per 10-μg/m^3^ increase in PM_1_, PM_2.5_, PM_10_, O_3_ and NO_2_) and the incidence of cardiovascular diseases among 7400 participants with cardiovascular–kidney–metabolic syndrome (CKM) stage 0**–**3: the results of time-varying Cox regression analysis. The figure presents results of the fully adjusted model: adjusted for age group, sex, residence, educational level, marital status, insurance, income, employment status, smoking status, sleep duration, and alcohol consumption.

**Figure 3:**
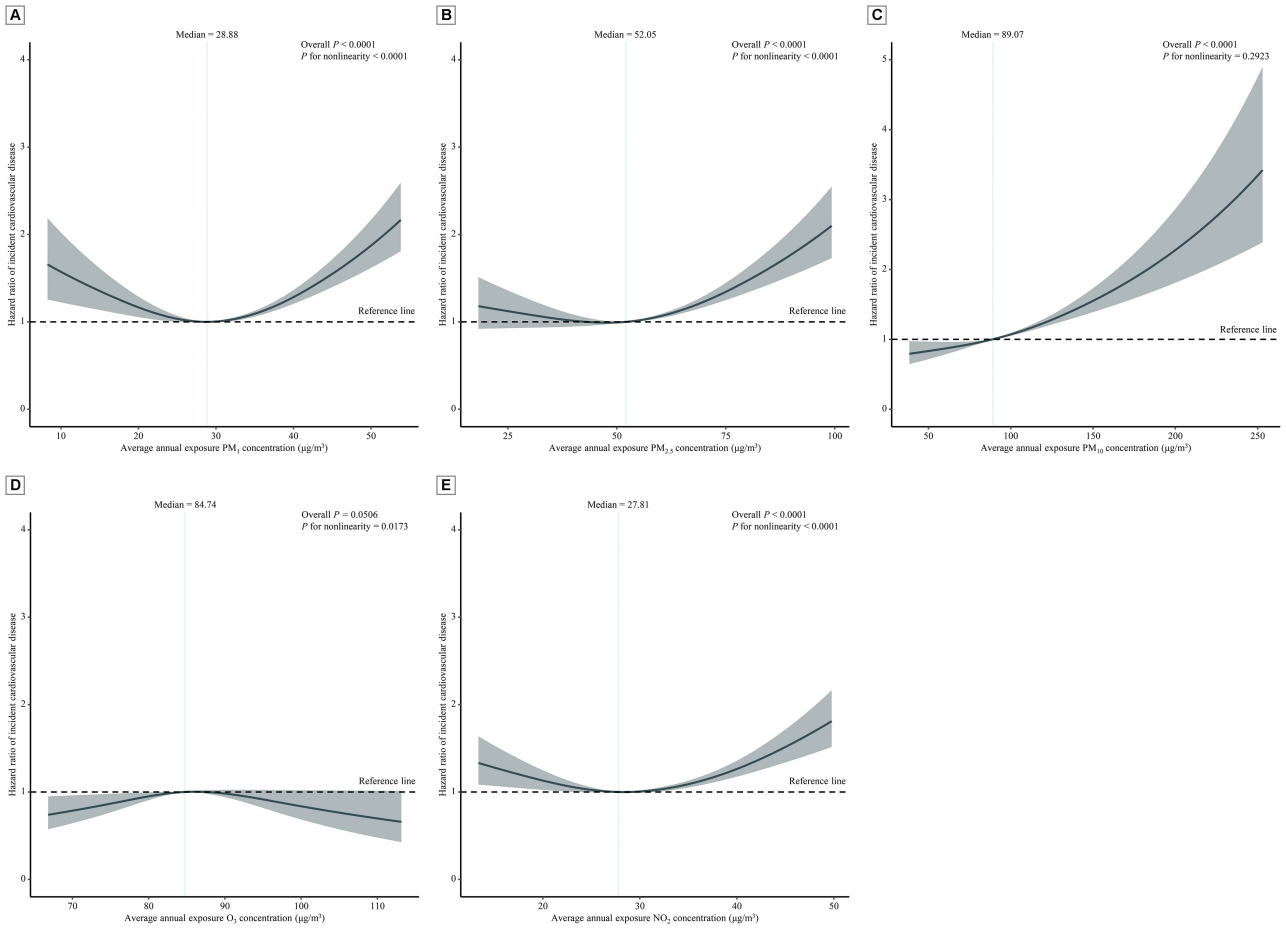
Exposure-response curves for the association of annual average exposure concentration in PM_1_ **(A)**, PM_2.5_ **(B)**, PM_10_ **(C)**, O_3_ **(D)**, and NO_2_ **(E)** with the incidence of cardiovascular diseases among 7400 participants with cardiovascular–kidney–metabolic syndrome (CKM) stages 0**–**3. Adjusted for age group, sex, residence, educational level, marital status, insurance, income group, cooking fuel, employment status, sleep duration, smoking status, and alcohol consumption. The solid blue line indicates the estimated effect, and the green shaded area represents the 95% confidence interval (CI). PM_1_, particle with aerodynamic diameter ≤1.0 μm; PM_2.5,_ particle with aerodynamic diameter ≤2.5 μm; PM_10_, particle with aerodynamic diameter ≤10 μm; O_3_, ozone; NO_2,_ nitrogen dioxide.

After the fully adjusted model (Model IV), each 10-μg/m^3^ increase in annual average concentration of PM_1_, PM_2.5_, PM_10_ and NO_2_ was significantly associated with 14.8% (HR 1.148, 95% CI 1.086–1.214), 9.6% (HR 1.096, 95% CI 1.064–1.128), 6.8% (HR 1.068, 95% CI 1.052–1.084), and 12.4% (HR 1.124, 95% CI 1.063–1.189) higher incidence risk of CVD among participants with CKM syndrome 0–3, respectively. The long-term exposure to ambient O_3_ concentration was positively associated with the increased incidence of CVD (HR = 1.025, 95% CI 0.962–1.091), but no statistically significant association was observed (**Figure 2**; **Supplementary Table 5**). We demonstrated that the positive nonlinear relationships between annual average exposures to PM_1_, PM_2.5_, O_3_ and NO_2_ with the elevated incident CVD (**Figure 3**, P for nonlinearity < 0.05), whereas a linear pattern across a broad PM_10_ concentration range of 38.3–252.7 µg/m^3^ (**Figure 3**, P for nonlinearity = 0.2923). After standardizing concentrations, PM_10_ showed the most responsible for elevated CVD risk, followed by PM_2.5_, PM_1_, NO_2_ and finally O_3_ (**Supplementary Table 6**).

**Figure 4:**
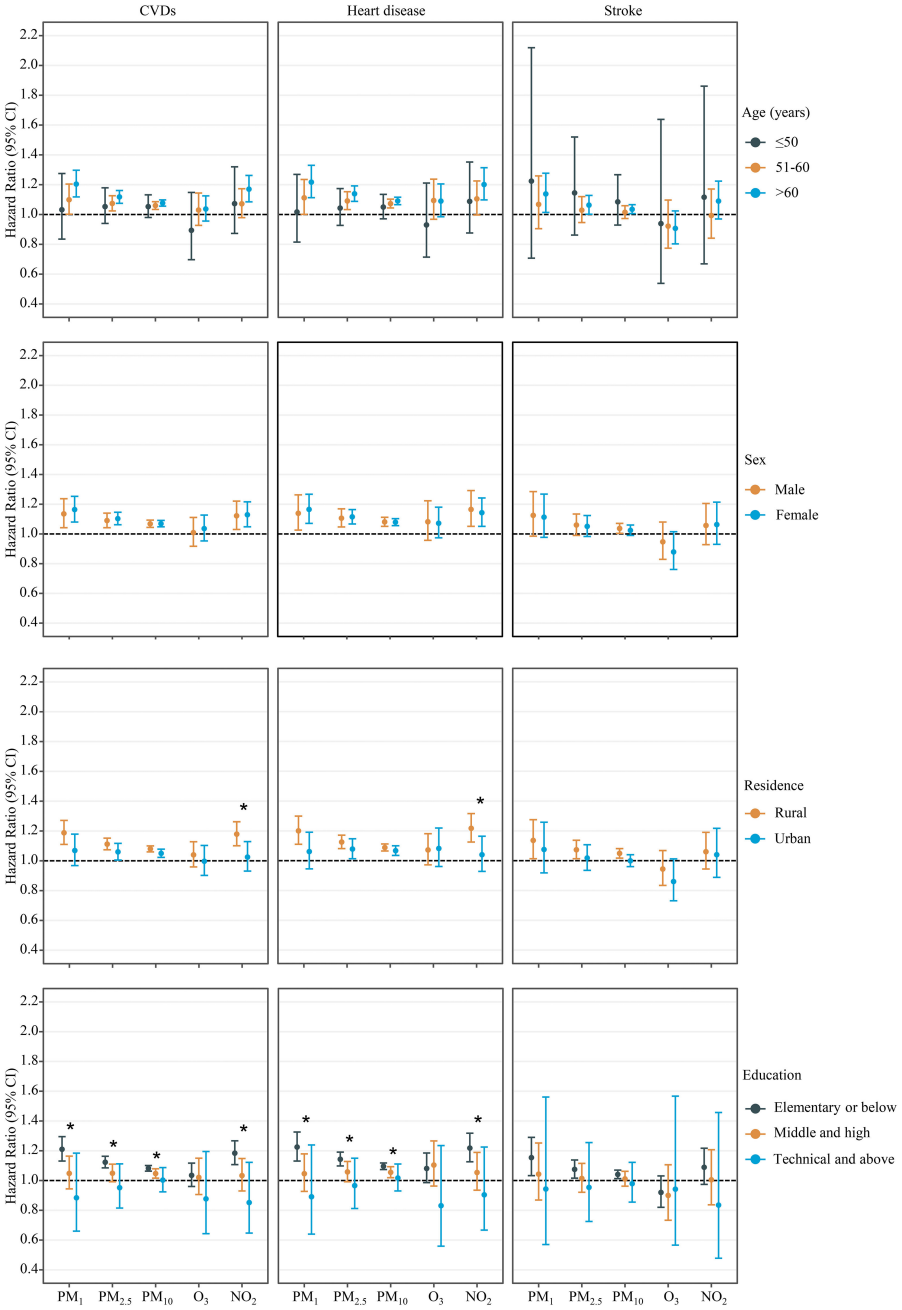
The association of per-10 μg/m^3^ increase in PM_1_, PM_2.5_, PM_10_, O_3_ and NO_2_ with the incidence of cardiovascular diseases and its major subtypes (heart disease and stroke), stratified by age group, sex, residence and education level. CI, confidence interval; PM_1_, particle with aerodynamic diameter ≤1.0 μm; PM_2.5,_ particle with aerodynamic diameter ≤2.5 μm; PM_10_, particle with aerodynamic diameter ≤10 μm; NO_2,_ nitrogen dioxide; O_3_, ozone. *interaction is statistically significant *P* < 0.05.

Subgroup and interaction analyses were conducted for various variables, including age groups, sex, residence, and education levels, to further investigate the relationship between air pollutants and incident CVD and its major subtypes (**Figure 4**; **Supplementary Tables 7**-**10**). In stratified analysis by age group, we found consistently and similarly elevated risks of developing from CVD, heart disease, and stroke associated with PM_1_, PM_2.5_, and PM_10_ among the different age subgroups, particularly in individuals aged 60 and older (**Figure 4**, P-interaction > 0.05). Regarding the effect modification by sex, we found generally stronger associations for PM_1_, PM_2.5_, PM_10_ and O_3_ with incidence risk of CVD among females compared with male participants, but without heterogeneous effects (**Figure 4**, P-interaction > 0.05). In addition, the association of PM_1_, PM_2.5_, PM_10_, O_3_ and NO_2_ with incident CVD and heart disease was greater in rural residents than in urban residents, with heterogeneous effects observed for NO_2_-associated risk of CVD (P-interaction = 0.022) and heart disease (P-interaction = 0.029). The level of education modified associations of incident CVD with PM_1_, PM_2.5_, PM_10_ and NO_2_, with these associations being adverse and generally stronger for participants reporting lower education level. Additionally, within subgroups stratified by CKM syndrome stage, the probability of developing CVD increased significantly with higher levels of air pollutant exposure, consistent with the overall findings (**Supplementary Table 11**).

### 3.3 Multi-pollutant model of associations between air pollution and incident CVD

In multiple-pollutant analyses, we observed more pronounced adverse associations between particulate matter (PM_1_, PM_2.5_, and PM_10_) and incidence risk of CVD after additionally adjusting for O_3_ and/or NO_2_ (**Table 2**). There was no statistically significant estimate for O_3_-related incidence risk of CVD, and a similar result persisted after adjustment for other pollutants, except for PM_10_. However, the significant correlations between the remaining pollutants (PM_1_, PM_2.5_, PM_10_, and NO_2_) and incident CVD persisted after adjusting for O_3_ exposure, with slightly increased adverse effects for each of the pollutants (**Table 2**).

**Table 2:** The association between per 10-μg/m^3^ increase in PM_1_, PM_2.5_, PM_10_, O_3_ and NO_2_ with incidence risk of cardiovascular diseases among cardiovascular-kidney-metabolic syndrome (CKM) stage 0–3 individuals – the results of time-varying cox-regression models with single and multiple pollutants.

Air pollutants exposure	Hazard ratio (95% CI)	P value
PM_1_	1.148 (1.086–1.214)	<0.001***
+O_3_	1.160 (1.095–1.230)	<0.001***
+NO_2_	1.173 (1.055–1.305)	0.003**
+O_3_+NO_2_	1.154 (1.031–1.292)	0.013*
PM_2.5_	1.096 (1.064–1.128)	<0.001***
+O_3_	1.105 (1.072–1.139)	<0.001***
+NO_2_	1.147 (1.091–1.206)	<0.001***
+O_3_+NO_2_	1.142 (1.085–1.203)	<0.001***
PM_10_	1.068 (1.052–1.084)	<0.001***
+O_3_	1.080 (1.063–1.098)	<0.001***
+NO_2_	1.092 (1.072–1.113)	<0.001***
+O_3_+NO_2_	1.096 (1.075–1.118)	<0.001***
O_3_	1.025 (0.962–1.091)	0.447
+PM_1_	0.963 (0.901–1.029)	0.260
+PM_2.5_	0.946 (0.886–1.011)	0.100
+PM_10_	0.893 (0.834–0.956)	0.001**
+NO_2_	0.934 (0.867–1.006)	0.071
+PM_1_+NO_2_	0.960 (0.887–1.040)	0.319
+PM_2.5_+NO_2_	0.972 (0.900–1.049)	0.469
+PM_10_+NO_2_	0.918 (0.854–0.986)	0.020*
NO_2_	1.124 (1.063–1.189)	<0.001***
+PM_1_	0.975 (0.876–1.085)	0.644
+PM_2.5_	0.902 (0.819–0.992)	0.034*
+PM_10_	0.889 (0.827–0.956)	0.001**
+O_3_	1.159 (1.085–1.238)	<0.001***
+PM_1_+O_3_	1.007 (0.887–1.144)	0.912
+PM_2.5_+O_3_	0.919 (0.821–1.028)	0.139
+PM_10_+O_3_	0.914 (0.846–0.987)	0.022*

The results were presented as hazard ratios and 95% CIs of CVD per-10 μg/m^3^ increase in annual air pollution exposure (PM_1_, PM_2.5_, PM_10_, O_3_ and NO_2_). Model 4, adjusted for age group, sex, residence, educational level, marital status, insurance, income group, cooking fuel, employment status, sleep duration, smoking status, and alcohol consumption. Abbreviations: PM_1_, particle with aerodynamic diameter ≤1.0 μm; PM_2.5_, particle with aerodynamic diameter ≤2.5 μm; PM_10_, particle with aerodynamic diameter ≤10 μm; O_3_, ozone; NO_2_, nitrogen dioxide. *P < 0.05; **P < 0.01; ***P < 0.001.

### 3.4 Sensitivity analysis

The sensitivity analyses largely supported the main result from the associations between air pollution exposure and incident CVD. Our findings were robust when excluding participants who developed CVD during the first two follow-up waves (**Supplementary Table 12**) and when applying a 2-year lag for annual pollutant concentrations (**Supplementary Table 13**), with similar magnitudes and statistical significance.

### 3.5 Mediation analysis

After adjustment for baseline covariates (age group, sex, education, residence, marital status, insurance, income, cooking fuel, employment, sleep duration, smoking and alcohol), the proportion of the pollution-CVD association mediated by metabolic syndrome was 4.26% for PM_1_ (*P* < 0.001), 3.11% for PM_2.5_ (*P* = 0.026), 2.39% for PM_10_ (*P* = 0.148), 2.98% for O_3_ (*P* = 0.004), and 5.62% for NO_2_ (*P* < 0.001), highlighting its significance in CVD risk assessment (**Table 3**).

**Table 3:** Mediation analysis of the effect of metabolic syndrome in the association between average air pollutants and the incidence of CVDs.

Air pollutants exposure (per 10 μg/m^3^)	Mediator	Total effect		Indirect effect		Direct effect		Proportion mediated, %
		Coefficient (95% CI)	P value	Coefficient (95% CI)	P value	Coefficient (95% CI)	P value	
PM_1_	Metabolic syndrome	-0.631 (-0.972 to -0.338)	<0.001	-0.027 (-0.051 to -0.009)	<0.001	-0.604 (-0.949 to -0.308)	<0.001	4.26 (1.31 to 10.19)
PM_2.5_		-0.448 (-0.639 to -0.275)	<0.001	-0.015 (-0.034 to -0.002)	0.026	-0.433 (-0.627 to -0.261)	<0.001	3.11 (0.40 to 8.02)
PM_10_		-0.325 (-0.428 to -0.228)	<0.001	-0.008 (-0.025 to -0.003)	0.148	-0.316 (-0.419 to -0.219)	<0.001	2.39 (-1.11 to 7.73)
O_3_		-0.430 (-0.911 to -0.027)	0.032	-0.013 (-0.027 to -0.003)	0.004	-0.417 (-0.901 to -0.018)	0.036	2.98 (0.49 to 16.38)
NO_2_		-0.486 (-0.802 to -0.214)	<0.001	-0.027 (-0.052 to -0.010)	<0.001	-0.458 (-0.774 to -0.185)	<0.001	5.62 (1.73 to 15.40)

PM_1_, particle with aerodynamic diameter ≤1.0 μm; PM_2.5,_ particle with aerodynamic diameter ≤2.5 μm; PM_10_, particle with aerodynamic diameter ≤10 μm; O_3_, ozone; NO_2,_ nitrogen dioxide.

The model was adjusted for age group, sex, educational level, residence (urban or rural), marital status, insurance, income group, cooking fuel, employment status, sleep duration, smoking status, and alcohol consumption at the baseline.

## 4 Discussion

In this large, nationally representative cohort study analyzing middle-aged and older adults with CKM syndrome (stages 0–3) using date from 28 provinces in mainland China, we investigated the independent and joint effects of long-term exposure to ambient air pollution on the risk of incident CVD and its major subtypes (heart disease and stroke), aiming to identify vulnerable populations. We suggested that individual exposure to PM_1_, PM_2.5_, PM_10_, and NO_2_ (per 10 μg/m^3^) was significantly associated to a 14.8%, 9.6%, 6.8%, and 12.4% elevated risk of developing CVD after full adjustment, respectively. In contrast, no significant association was observed between O_3_ exposure and incident CVD among Chinese middle and older adults with CKM syndrome stages 0–3. Of note, PM_10_ exhibited the most responsible for the increased incidence risk of CVD after standardizing annual concentrations, followed by PM_2.5_, PM_1_, NO_2_ and O_3_. In addition, stronger adverse associations of PM_1_, PM_2.5_, PM_10_, and NO_2_ were identified for incident CVD among CKM syndrome participants with lower educational attainment compared with their counterparts, and heterogeneous effects were observed for NO_2_-associated CVD and heart disease incidence in rural residents, whereas no such differential effects were detected for the other air pollutants across subgroups. Finally, we found generally stronger adverse associations between particulate matter (PM_1_, PM_2.5_, and PM_10_) and incident CVD after additionally adjusting for O_3_ and/or NO_2_. The results largely remained consistent with the main findings in sensitivity analyses.

As the largest low- and middle-income country, China has suffered from significantly higher annual average concentrations of PM_2.5_, PM_10_, O_3_ and NO_2_ than the levels recommended by WHO, particularly in northwestern region, driven by rapid urbanization, traffic, and industrial emissions (7). Subsequently, our study found that participants with CKM syndrome who developed CVD had a significantly higher level of exposure to ambient air pollutants compared with those without incident CVD (8, 13). Since 2013, the Chinese government has implemented a series of control policies under the Air Pollution Prevention and Control Action Plan, resulting in significant reductions in air pollutant levels both nationally and regionally (42). Nevertheless, the ambient concentration level of O_3_ has risen steadily, gradually replacing particulate matter as the main source of pollution in many population-densely megacities in China during the warm-season (11).

We suggested that long-term ambient air pollution exposure has adverse impacts on the new-onset or development of CVD, which is consistent with previous studies (7–10, 12, 20). A recent large-scale prospective cohort study involving over 168,010 UK Biobank participants with pre-hypertension reported that greater exposure to air pollutants was positively associated with a higher incidence of CVD (PM_2.5_: HR 1.045 [95% CI 1.022-1.068]; PM_10_: HR 1.017 [95% CI 0.995-1.039]; NO_2_: HR 1.028 [95% CI 1.005-1.050]; NO_x_: HR 1.033 [95% CI 1.014-1.053]) (13). However, Chinese CKM syndrome participants higher CVD risk than those in UK, where ambient air pollution exposure general <30 μg/m^3^ (13). One important reason may be that air pollution in the UK is less severe than in China and that patients with CKM show higher susceptibility. In addition, a Chinese national cohort study indicated that for each 10-μg/m^3^ increase in the 16-year average concentration of PM_2.5_, the relative risk for CVD incidence and mortality significantly increased by 25.1% (HR 1.251, 95% CI 1.220-1.283) and 16.4% (HR 1.164, 95% CI 1.117-1.213), respectively (14). The methodological differences in analysis could potentially explain this disparity. Liang et al. (14) adopted average PM_2.5_ exposure over the 16 years (2000–2015) before death or end of follow-up instead of time-varying exposures, which may overestimate impacts, as particulate air pollution has decreased during more recent follow-up intervals in China (42). Several mechanisms underlying air pollution–mediated systemic CVD risk have been proposed and can be encapsulated into four primary initiating pathways (systemic inflammation, oxidative stress, accelerated atherosclerosis, and alteration of cardiac autonomic function) (14) and 6 secondary mediators pathways (10). Exploring the mediating role of metabolic syndrome in the association between air pollution exposure and incident CVD could facilitate more precise identification of high-risk populations and the subsequent enhancement of clinical interventions. Furthermore, recent evidence has shown that metabolic dysfunction-associated steatotic liver disease (MASLD) is associated with subclinical cardiac dysfunction, including impaired left ventricular global longitudinal strain, supporting its potential role as an early cardiometabolic risk phenotype (43).

We observed a lack of statistically significant association between O_3_ and the risk of developing CVD in our cohort study, which contrasts with some studies focused on the general population (7, 11, 22). For instance, Zhu and colleagues (11) reported that long-term warm-season O_3_ (per 10 μg/m^3^ increase) was associated with a 7.8% greater risk of incident overall CVD (HR 1.078, 95% CI 1.050–1.106) in 36948 participants from China. However, the exact mechanisms by which O_3_ offsets or masks the adverse effects on incident CVD in populations with CKM syndrome are not fully understood and need to be further elucidated in future research. Notably, individuals who developed adverse events during follow-up had no differences in exposure to O_3_ at baseline compared with non-CVD participants. The absence of a significant association for O_3_ in our multi-pollutant analysis may be explained by the dominant effects of particulate matter (PM_1_, PM_2.5_, and PM_10_) and NO_2_, which accounted for a substantial proportion of the shared variance in cardiovascular risk. Furthermore, previous studies suggest that O_3_ could potentially have antimicrobial and anti-inflammatory effects (44, 45), or show a nonlinear (U-shaped) exposure–response relationship with health (46), indicating that moderate exposure to O_3_ may not significantly contribute to chronic conditions.

Notably, the nonlinear exposure–response relationships identified for PM_1_, PM_2.5_, O_3_, and NO_2_, indicating the presence of specific exposure thresholds, underscore substantial relevance for the development of air quality standards and the evaluation of disease burden (7, 30). In 2021, the WHO updated its global air quality guidelines in response to accumulating epidemiological and toxicological evidence, which demonstrates that adverse cardiovascular outcomes may still occur even at relatively low concentration levels of fine particulate matter. The identification of these thresholds is particularly significant in the context of participants with CKM syndrome stages 0–3, who may experience a more pronounced cardiovascular risk with slight increases in pollutant levels beyond these thresholds. This observation suggests that traditional pollution metrics, which often assume a linear relationship between exposure and CVD outcomes, may be overly simplistic when applied to vulnerable populations. At such low exposure levels, the mild oxidative stress induced by O_3_ exposure might stimulate cytoprotective pathways, including the Nrf2-mediated antioxidant defense mechanism, or modulate the immune system (47), which could potentially alleviate acute cardiovascular injury (48).

The present study has some limitations that should be considered. First, air pollution exposure was calculated at the county level rather than by participants’ residential addresses, which might lead to ambient air pollutant exposure misclassification. Second, several important potential confounders were not widely available in the CHARLS database, including physical activity, medication use, dietary intake (e.g., protein, fat, cooking oil, salt, and sugar intake), and other lifestyle characteristics, which might contribute to CVD in high-risk populations with CKM syndrome stages 0–3. Third, we conducted only one baseline (2011 wave) assessment of CKM syndrome stage without accounting for longitudinal progression. Finally, the primary outcome of CVD was ascertained by questionnaire during each follow-up survey wave from 2011 to 2018, which may involve recall and self-report bias.

## 5 Conclusions

In this longitudinal study of 7400 Chinese middle-aged and older adults with CKM syndrome stages 0–3 from CHARLS, each 10 μg/m^3^ increase in PM_1_, PM_2.5_, PM_10_, and NO_2_ was associated with 14.8%, 9.6%, 6.8%, and 12.4% higher incidence of CVD, respectively, whereas O_3_ exhibited no significant relationship. After standardizing exposures, PM_10_ exhibited the largest relative association with incident CVD, highlighting the importance for targeted interventions for vulnerable subgroups, especially for those who received low educational level. Future research is required to further explore these associations and provide more evidence regarding the driving factors of ambient air pollution in the dynamic trajectories of CVD to inform targeted healthcare policies and preventive measures.

## Data Availability

Publicly available datasets were analyzed in this study. This data can be found here: https://charls.pku.edu.cn/.
