# Supplementary material for: Association between air pollution and incident cardiovascular diseases among a population with cardiovascular-kidney-metabolic syndrome stages 0–3: the first evidence from the China Health and Retirement Longitudinal Study
Source: Front Endocrinol (Lausanne). 2026 Jun 23;17:1852623. doi: 10.3389/fendo.2026.1852623 (PMC13337506; doi:10.3389/fendo.2026.1852623)
Supplement: Supplementary file 1 [file SupplementaryFile1.docx]

**Supplementary materials**

Association between air pollution and incident cardiovascular diseases among a population with cardiovascular-kidney-metabolic syndrome stages 0–3: the first evidence from the China Health and Retirement Longitudinal Study

[**Supplementary Table S1.** Assessment of Cardiovascular-kidney-metabolic (CKM) syndrome stages 0-3 in CHARLS^a^. 3](#_Toc226739837)

[**Supplementary Table S2.** The 10-fold cross-validation *R^2^* and Root-Mean-Square Error of estimation of air pollutants. 4](#_Toc226739838)

[**Supplementary Table S3.** Spearman correlation coefficients of average air pollutant concentrations during the follow up. 5](#_Toc226739839)

[**Supplementary Table S4.** Descriptive statistics levels of annual average air pollution for 7,400 cardiovascular-kidney-metabolic (CKM) syndrome stages 0-3 participants during the follow up. 6](#_Toc226739840)

[**Supplementary Table S5.** The associations between per 10 μg/m^3^ increase in PM_1_, PM_2.5_, PM_10_, O_3_, NO_2_ and incidence of cardiovascular disease in 7,400 cardiovascular-kidney-metabolic (CKM) syndrome stages 0-3 participants: the results of time-varying cox regression analysis. 7](#_Toc226739841)

[**Supplementary Table S6.** The associations between the standardized exposure to air pollutants concentrations (PM_1_, PM_2.5_, PM_10_, O_3_, and NO_2_) and the incidence of cardiovascular disease in 7,400 cardiovascular-kidney-metabolic (CKM) syndrome stages 0-3 participants: the results of time-varying cox regression analysis. 8](#_Toc226739842)

[**Supplementary Table S7.** The association between per 10 μg/m^3^ increase in PM_1_, PM_2.5_, PM_10_, O_3_, NO_2_ and the incidence of cardiovascular diseases, and its major subtypes (heart disease and stroke) stratified by age group. 9](#_Toc226739843)

[**Supplementary Table S8.** The association between per 10 μg/m^3^ increase in PM_1_, PM_2.5_, PM_10_, O_3_, NO_2_ and incidence of cardiovascular diseases, and its major subtypes (heart disease and stroke) stratified by sex. 12](#_Toc226739844)

[**Supplementary Table S9.** The association between per 10 μg/m^3^ increase in PM_1_, PM_2.5_, PM_10_, O_3_, NO_2_ and incidence of cardiovascular diseases, and its major subtypes (heart disease and stroke) stratified by residence. 14](#_Toc226739845)

[**Supplementary Table S10.** The association between per 10 μg/m^3^ increase in PM_1_, PM_2.5_, PM_10_, O_3_, NO_2_ and incidence of cardiovascular diseases, and its major subtypes (heart disease and stroke) stratified by education level. 16](#_Toc226739846)

[**Supplementary Table S11**. The associations between per 10 μg/m^3^ increase in PM_1_, PM_2.5_, PM_10_, O_3_, NO_2_ and incidence of cardiovascular diseases across cardiovascular-kidney-metabolic (CKM) syndrome stages: the results of time-varying cox regression analysis. 19](#_Toc226739847)

[**Supplementary Table S12.** The associations between per 10 μg/m^3^ increase in PM_1_, PM_2.5_, PM_10_, O_3_, NO_2_ and incidence of cardiovascular diseases in 6,962 cardiovascular-kidney-metabolic (CKM) syndrome stages 0-3 participants: the results of sensitivity analysis where participants developing cardiovascular diseases in the first two follow-up waves were excluded. 20](#_Toc226739848)

[**Supplementary Table S13**. The associations between per 10 μg/m^3^ increase in PM_1_, PM_2.5_, PM_10_, O_3_, NO_2_ and incidence of cardiovascular diseases in 7,400 cardiovascular-kidney-metabolic (CKM) syndrome stages 0-3 participants: the results of time-varying cox regression analysis (lag effects were adjusted for 2-years). 21](#_Toc226739849)

[**Supplementary Figure S1.** City-level annual concentration exposure distribution maps of air pollutants for PM_1_, PM_2.5_, PM_10_, O_3_, and NO_2_ in China. 22](#_Toc226739850)

[**Supplementary Figure S2.** Average annual concentration of air pollutants in PM_1_, PM_2.5_, PM_10_, O_3_, and NO_2_ by cardiovascular disease status (median, interquartile range) during their follow-up. 23](#_Toc226739851)

**Supplementary Table S1.** Assessment of Cardiovascular-kidney-metabolic (CKM) syndrome stages 0-3 in CHARLS^a^.

| Stage 0 | Participants with normal body mass index (BMI <23 kg/m^2^), and normal waist circumference (<80 and <90 cm for women and men, respectively). |
| --- | --- |
| Stage 1 | Participants with elevated body mass index (BMI ≥23kg/m^2^), elevated waist circumference (≥80 and 90 cm for women and men, respectively), or prediabetes (defined as a fasting blood glucose ≥100-126 mg/dL or glycated hemoglobin [HbA1c] ≥5.7%-6.5%. |
| Stage 2 | Individuals with metabolic risk factors or moderate-to-high risk chronic kidney disease (CKD)^b^.  Diabetes: fasting blood glucose ≥ 126 mg/dL or HbA1c ≥6.5%, and/or a self-reported physician-diagnosed, and/or taking hypoglycemic medications;  Hypertension: a self-reported physician-diagnosed, and/or any antihypertensive medication use, and/or an average systolic/diastolic blood pressure (SBP/DBP) ≥130/80 mmHg;  Metabolic syndrome: Meet ≥3 of the following: (1) elevated waist circumference; (2) prediabetes; (3) elevated blood pressure SBP ≥130, DBP ≥80 mmHg, and/or self-reported physician-diagnosed with hypertension, and/or receiving pressure-lowering treatment; (4) elevated fasting serum triglycerides (≥135 mg/dL); (5) low level of high density lipoprotein (HDL) cholesterol <40 mg/dL or <50 mg/dL for men or women;  CKD: self-reported history of CKD or estimated glomerular filtration rate (eGFR) ≥30-60 ml/min/1.73m^2^. |
| Stage 3 | Individuals identified with subclinical cardiovascular disease (eGFR < 30 ml/min/1.73m^2^ or high-predicted 10-year cardiovascular disease risk)^c^. |

^a^ All participants were Asian ethnicity in CHARLS database. CKM syndrome stages were identified in accordance with the 2023 American Heart Association (AHA) Presidential Advisory on CKM Health.(1)

^b^ CKD were identified based on self-reported CKD and eGFR. The eGFR was calculated using the race-free Chronic Kidney Disease Epidemiology Collaboration 2021 creatinine equation recommended by AHA.(2)

^c^ 10-year cardiovascular disease (CVD defined as heart problems and stroke in CHARLS) risk was estimated with the AHA Predicting Risk of CVD EVENTs (PREVENT) equations.(3) High-predicted risk was defined as ≥20% 10-year CVD risk (based on recommended thresholds [<https://professional.heart.org/en/guidelines-and-statements/prevent-calculator>]). The PREVENT equations were developed and validated for adults 30-79 years of age. As such, risk was not estimated for adults <30 years. However, to minimize underestimation of CKD Stage 3, adults ≥80 years were not excluded from 10-year CVD risk. Instead, adults ≥80 years were assigned an age of 79 years when determining 10-year CVD risk to allow for conservative estimates. Further, PREVENT was developed for variables with the following ranges: total cholesterol 130-320 mg/dL, HDL cholesterol 20-100 mg/dL, SBP 90-200 mmHg, BMI 18.5-39.9, and eGFR 15-140 mL/min/1.73m². To approximate PREVENT risk strata, values for these variables above or below these bounds were set to the upper or lower bounds of allowable values respectively (for example, total cholesterol of 350 mg/dL was set as 320 mg/dL and HDL cholesterol of 19 was set as 20mg/dL), which was full described previous.(4)

**Supplementary Table S2.** The 10-fold cross-validation *R^2^* and Root-Mean-Square Error of estimation of air pollutants.

| **Air pollution** | PM_1_ | PM_2.5_ | PM_10_ | O_3_ | NO_2_ |
| --- | --- | --- | --- | --- | --- |
| *R^2^* | 0.83 | 0.92 | 0.90 | 0.89 | 0.84 |
| Root-Mean-Square Error | 9.50μg/m^3^ | 10.76µg/m^3^ | 21.12µg/m^3^ | 15.77µg/m^3^ | 7.99μg/m^3^ |

Abbreviations: PM_1_, particle with aerodynamic diameter ≤1.0 μm; PM_2.5,_ particle with aerodynamic diameter ≤2.5 μm; PM_10_, particle with aerodynamic diameter ≤10 μm; O_3_, ozone; NO_2,_ nitrogen dioxide.

**Supplementary Table S3.** Spearman correlation coefficients of average air pollutant concentrations during the follow up.

| **Air pollution** | PM_1_ | PM_2.5_ | PM_10_ | O_3_ | NO_2_ |
| --- | --- | --- | --- | --- | --- |
| PM_1_ | 1 |  |  |  |  |
| PM_2.5_ | 0.973 *** | 1 |  |  |  |
| PM_10_ | 0.872 *** | 0.933 *** | 1 |  |  |
| O_3_ | 0.538 *** | 0.524 *** | 0.596 *** | 1 |  |
| NO_2_ | 0.891 *** | 0.860 *** | 0.809 *** | 0.696 *** | 1 |

Abbreviations: PM_1_, particle with aerodynamic diameter ≤1.0 μm; PM_2.5,_ particle with aerodynamic diameter ≤2.5 μm; PM_10_, particle with aerodynamic diameter ≤10 μm; O_3_, ozone; NO_2,_ nitrogen dioxide; * *P*<0.05; ** *P*<0.01; *** *P*<0.001.

# **Supplementary Table S4.** Descriptive statistics levels of annual average air pollution for 7,400 cardiovascular-kidney-metabolic (CKM) syndrome stages 0-3 participants during the follow up.

| **Air pollution** | **Mean** | **SD** | **P25** | **P50** | **P75** | **IQR** | **>WHO interim target ^a^, n (%)** | **>WHO AQG level ^b^, n (%)** |
| --- | --- | --- | --- | --- | --- | --- | --- | --- |
| PM_1_ (μg/m^3^) ^c^ | 29.75 | 9.21 | 22.13 | 28.68 | 36.23 | 14.10 | NA | NA |
| PM_2.5_ (μg/m^3^) | 53.15 | 17.00 | 38.57 | 51.51 | 65.48 | 26.91 | 7,400 (100%) | 7,400 (100%) |
| PM_10_ (μg/m^3^) | 90.83 | 31.05 | 61.89 | 88.23 | 113.09 | 51.20 | 7,400 (100%) | 7,400 (100%) |
| O_3_ (μg/m^3^) | 86.61 | 7.47 | 80.25 | 85.34 | 92.40 | 12.14 | 7,400 (100%) | 7,400 (100%) |
| NO_2_ (μg/m^3^) | 29.70 | 9.34 | 22.06 | 27.91 | 36.32 | 14.26 | 6,273 (84.8%) | 7,400 (100%) |

WHO, World Health Organization; AQG, air quality guideline; PM_1_, particle with aerodynamic diameter ≤1.0 μm; PM_2.5,_ particle with aerodynamic diameter ≤2.5 μm; PM_10_, particle with aerodynamic diameter ≤10 μm; O_3_, ozone; NO_2,_ nitrogen dioxide; SD, standard deviation, IQR, interquartile range, NA, not applicable.

^a^ The WHO interim targets for air pollutants are 10 μg/m^3^, 20 μg/m^3^, 70 μg/m^3^ and 20 μg/m^3^ for PM_2.5_, PM_10_, O_3_, and NO_2_, respectively.(5)

^b^ The WHO 2021 AQG levels are 5 μg/m^3^, 15 μg/m^3^, 60 μg/m^3^, and 10 μg/m^3^ for PM_2.5_, PM_10_, O_3_ and NO_2_, respectively.(5)

^c^ The limit values for PM_1_ are not stated in the WHO or UK air quality guidelines.

# **Supplementary Table S5.** The associations between per 10 μg/m^3^ increase in PM_1_, PM_2.5_, PM_10_, O_3_, NO_2_ and incidence of cardiovascular disease in 7,400 cardiovascular-kidney-metabolic (CKM) syndrome stages 0-3 participants: the results of time-varying cox regression analysis.

| Air pollutants exposure | Hazzard ratio (95% CI) |  |  |  |
| --- | --- | --- | --- | --- |
|  | Model I ^a^ | Model II ^b^ | Model III ^c^ | Model IV ^d^ |
| PM_1_ | 1.140 (1.082-1.200) *** | 1.146 (1.088-1.208) *** | 1.147 (1.085-1.213) *** | 1.148 (1.086-1.214) *** |
| PM_2.5_ | 1.092 (1.063-1.122) *** | 1.095 (1.066-1.125) *** | 1.095 (1.064-1.127) *** | 1.096 (1.064-1.128) *** |
| PM_10_ | 1.068 (1.053-1.084) *** | 1.071 (1.056-1.087) *** | 1.068 (1.052-1.084) *** | 1.068 (1.052-1.084) *** |
| O_3_ | 1.014 (0.955-1.078) | 1.022 (0.962-1.085) | 1.026 (0.964-1.092) | 1.025 (0.962-1.091) |
| NO_2_ | 1.114 (1.058-1.174) *** | 1.123 (1.065-1.185) *** | 1.124 (1.063-1.188) *** | 1.124 (1.063-1.189) *** |

Abbreviations: PM_1_, particle with aerodynamic diameter ≤1.0 μm; PM_2.5,_ particle with aerodynamic diameter ≤2.5 μm; PM_10_, particle with aerodynamic diameter ≤10 μm; O_3_, ozone; NO_2,_ nitrogen dioxide.

^a^ Model I, unadjusted;

^b^ Model II, adjusted for age group and sex;

^c^ Model III, adjusted for age group, sex, educational level, residence (urban or rural), marital status, insurance, income group, cooking fuel, employment status, and sleep duration;

^d^ Model IV, adjusted for age group, sex, educational level, residence (urban or rural), marital status, insurance, income group, cooking fuel, employment status, sleep duration, smoking status, and alcohol consumption.

* *P*<0.05; ** *P*<0.01; *** *P*<0.001.

# **Supplementary Table S6.** The associations between the standardized exposure to air pollutants concentrations (PM_1_, PM_2.5_, PM_10_, O_3_, and NO_2_) and the incidence of cardiovascular disease in 7,400 cardiovascular-kidney-metabolic (CKM) syndrome stages 0-3 participants: the results of time-varying cox regression analysis.

| Air pollutants exposure | Hazzard ratio (95% CI) |  |  |  |
| --- | --- | --- | --- | --- |
|  | Model I ^a^ | Model II ^b^ | Model III ^c^ | Model IV ^d^ |
| PM_1_ | 1.136 (1.080-1.195) *** | 1.143 (1.085-1.203) *** | 1.143 (1.083-1.207) *** | 1.144 (1.084-1.208) *** |
| PM_2.5_ | 1.173 (1.117-1.231) *** | 1.179 (1.122-1.239) *** | 1.178 (1.118-1.242) *** | 1.180 (1.120-1.243) *** |
| PM_10_ | 1.237 (1.182-1.295) *** | 1.249 (1.193-1.307) *** | 1.236 (1.178-1.297) *** | 1.237 (1.179-1.298) *** |
| O_3_ | 1.014 (0.957-1.074) | 1.020 (0.964-1.080) | 1.025 (0.966-1.087) | 1.023 (0.964-1.086) |
| NO_2_ | 1.108 (1.054-1.164) *** | 1.116 (1.062-1.174) *** | 1.117 (1.059-1.177) *** | 1.117 (1.059-1.177) *** |

Abbreviations: PM_1_, particle with aerodynamic diameter ≤1.0 μm; PM_2.5,_ particle with aerodynamic diameter ≤2.5 μm; PM_10_, particle with aerodynamic diameter ≤10 μm; O_3_, ozone; NO_2,_ nitrogen dioxide.

^a^ Model I, unadjusted;

^b^ Model II, adjusted for age group and sex;

^c^ Model III, adjusted for age group, sex, educational level, residence (urban or rural), marital status, insurance, income group, cooking fuel, employment status, and sleep duration;

^d^ Model IV, adjusted for age group, sex, educational level, residence (urban or rural), marital status, insurance, income group, cooking fuel, employment status, sleep duration, smoking status, and alcohol consumption.

* *P*<0.05; ** *P*<0.01; *** *P*<0.001.

| **Supplementary Table S7.** The association between per 10 μg/m^3^ increase in PM_1_, PM_2.5_, PM_10_, O_3_, NO_2_ and the incidence of cardiovascular diseases, and its major subtypes (heart disease and stroke) stratified by age group. | | | | | |
| --- | --- | --- | --- | --- | --- |
| Air pollutants exposure | Variable | Hazzard ratio (95% CI) |  |  |  |
|  |  | Model I ^a^ | Model II ^b^ | Model III ^c^ | Model IV ^d^ |
| Cardiovascular disease | | | | | |
| PM_1_ |  |  |  |  |  |
|  | ≤50  51-60  >60 | 1.044 (0.859-1.268) | 1.046 (0.861-1.272) | 1.033 (0.836-1.276) | 1.032 (0.835-1.275) |
|  |  | 1.112 (1.017-1.214) * | 1.110 (1.016-1.213) * | 1.097 (1.000-1.203) * | 1.099 (1.002-1.204) * |
|  |  | 1.188 (1.109-1.273) *** | 1.188 (1.108-1.273) *** | 1.204 (1.117-1.297) *** | 1.204 (1.118-1.297) *** |
| PM_2.5_ |  |  |  |  |  |
|  | ≤50 | 1.061 (0.960-1.173) | 1.063 (0.961-1.175) | 1.054 (0.942-1.180) | 1.053 (0.940-1.179) |
|  | 51-60 | 1.079 (1.030-1.129) ** | 1.078 (1.030-1.128) ** | 1.072 (1.022-1.125) ** | 1.074 (1.024-1.126) ** |
|  | >60 | 1.112 (1.073-1.153) *** | 1.112 (1.072-1.152) *** | 1.117 (1.075-1.161) *** | 1.118 (1.075-1.161) *** |
| PM_10_ |  |  |  |  |  |
|  | ≤50 | 1.059 (0.995-1.126) | 1.060 (0.997-1.128) | 1.054 (0.981-1.132) | 1.053 (0.980-1.132) |
|  | 51-60 | 1.063 (1.039-1.089) *** | 1.063 (1.038-1.088) *** | 1.059 (1.033-1.085) *** | 1.060 (1.034-1.086) *** |
|  | >60 | 1.078 (1.059-1.099) *** | 1.078 (1.058-1.099) *** | 1.077 (1.056-1.098) *** | 1.077 (1.056-1.098) *** |
| O_3_ |  |  |  |  |  |
|  | ≤50 | 0.908 (0.716-1.153) | 0.910 (0.718-1.154) | 0.892 (0.695-1.144) | 0.894 (0.697-1.148) |
|  | 51-60 | 1.049 (0.948-1.162) | 1.048 (0.947-1.160) | 1.029 (0.927-1.143) | 1.030 (0.927-1.144) |
|  | >60 | 1.024 (0.947-1.107) | 1.021 (0.944-1.104) | 1.039 (0.958-1.127) | 1.037 (0.956-1.125) |
| NO_2_ |  |  |  |  |  |
|  | ≤50 | 1.072 (0.887-1.295) | 1.076 (0.891-1.300) | 1.072 (0.872-1.319) | 1.073 (0.873-1.320) |
|  | 51-60 | 1.093 (1.001-1.193) * | 1.093 (1.001-1.193) * | 1.068 (0.976-1.170) | 1.072 (0.979-1.173) |
|  | >60 | 1.154 (1.074-1.239) *** | 1.151 (1.073-1.236) *** | 1.171 (1.086-1.263) *** | 1.170 (1.085-1.262) *** |
| Heart disease | | | | | |
| PM_1_ |  |  |  |  |  |
|  | ≤50 | 0.997 (0.807-1.232) | 1.001 (0.809-1.238) | 1.019 (0.816-1.271) | 1.017 (0.815-1.269) |
|  | 51-60  >60 | 1.136 (1.026-1.257) * | 1.133 (1.024-1.255) * | 1.108 (0.998-1.232) | 1.112 (1.001-1.235) * |
|  |  | 1.201 (1.106-1.304) *** | 1.199 (1.105-1.302) *** | 1.217 (1.114-1.330) *** | 1.217 (1.113-1.330) *** |
| PM_2.5_ |  |  |  |  |  |
|  | ≤50 | 1.036 (0.929-1.154) | 1.038 (0.931-1.158) | 1.045 (0.929-1.175) | 1.043 (0.927-1.174) |
|  | 51-60 | 1.101 (1.045-1.160) *** | 1.100 (1.044-1.159) *** | 1.089 (1.031-1.151) ** | 1.091 (1.033-1.153) ** |
|  | >60 | 1.131 (1.084-1.181) *** | 1.130 (1.083-1.179) *** | 1.139 (1.088-1.192) *** | 1.139 (1.088-1.192) *** |
| PM_10_ |  |  |  |  |  |
|  | ≤50 | 1.046 (0.976-1.122) | 1.049 (0.978-1.124) | 1.051 (0.973-1.136) | 1.050 (0.971-1.135) |
|  | 51-60 | 1.079 (1.051-1.108) *** | 1.078 (1.051-1.107) *** | 1.072 (1.043-1.102) *** | 1.073 (1.044-1.103) *** |
|  | >60 | 1.091 (1.068-1.115) *** | 1.091 (1.067-1.115) *** | 1.091 (1.067-1.117) *** | 1.091 (1.066-1.116) *** |
| O_3_ |  |  |  |  |  |
|  | ≤50 | 0.921 (0.703-1.206) | 0.924 (0.706-1.210) | 0.927 (0.712-1.207) | 0.930 (0.714-1.211) |
|  | 51-60 | 1.105 (0.982-1.244) | 1.103 (0.980-1.241) | 1.093 (0.968-1.235) | 1.094 (0.968-1.237) |
|  | >60 | 1.066 (0.968-1.173) | 1.060 (0.963-1.167) | 1.092 (0.988-1.207) | 1.090 (0.985-1.205) |
| NO_2_ |  |  |  |  |  |
|  | ≤50 | 1.054 (0.856-1.297) | 1.062 (0.862-1.308) | 1.087 (0.876-1.351) | 1.088 (0.876-1.352) |
|  | 51-60 | 1.130 (1.024-1.247) * | 1.130 (1.023-1.247) * | 1.101 (0.993-1.220) | 1.105 (0.997-1.225) |
|  | >60 | 1.181 (1.085-1.286) *** | 1.177 (1.082-1.281) *** | 1.203 (1.100-1.316) *** | 1.201 (1.098-1.314) *** |
| Stroke | | | | | |
| PM_1_ |  |  |  |  |  |
|  | ≤50 | 1.343 (0.861-2.094) | 1.328 (0.860-2.051) | 1.214 (0.706-2.089) | 1.224 (0.707-2.119) |
|  | 51-60  >60 | 1.030 (0.881-1.203) | 1.031 (0.883-1.204) | 1.066 (0.904-1.257) | 1.068 (0.905-1.259) |
|  |  | 1.127 (1.010-1.257) * | 1.127 (1.010-1.257) * | 1.136 (1.012-1.275) * | 1.138 (1.014-1.277) * |
| PM_2.5_ |  |  |  |  |  |
|  | ≤50 | 1.199 (0.956-1.503) | 1.191 (0.954-1.485) | 1.142 (0.863-1.510) | 1.145 (0.862-1.520) |
|  | 51-60 | 1.010 (0.932-1.094) | 1.011 (0.933-1.095) | 1.028 (0.945-1.119) | 1.029 (0.946-1.120) |
|  | >60 | 1.063 (1.005-1.124) * | 1.063 (1.005-1.124) * | 1.062 (1.001-1.127) * | 1.063 (1.002-1.128) * |
| PM_10_ |  |  |  |  |  |
|  | ≤50 | 1.115 (0.996-1.250) | 1.111 (0.993-1.243) | 1.083 (0.930-1.262) | 1.085 (0.929-1.267) |
|  | 51-60 | 1.006 (0.964-1.049) | 1.006 (0.964-1.049) | 1.014 (0.972-1.058) | 1.015 (0.973-1.059) |
|  | >60 | 1.040 (1.011-1.069) ** | 1.040 (1.011-1.069) ** | 1.035 (1.005-1.065) * | 1.035 (1.006-1.066) * |
| O_3_ |  |  |  |  |  |
|  | ≤50 | 0.977 (0.655-1.457) | 0.967 (0.650-1.437) | 0.930 (0.538-1.606) | 0.939 (0.538-1.638) |
|  | 51-60 | 0.945 (0.795-1.124) | 0.946 (0.796-1.125) | 0.920 (0.772-1.095) | 0.922 (0.774-1.097) |
|  | >60 | 0.919 (0.818-1.033) | 0.920 (0.818-1.034) | 0.907 (0.804-1.024) | 0.907 (0.803-1.024) |
| NO_2_ |  |  |  |  |  |
|  | ≤50 | 1.216 (0.806-1.834) | 1.197 (0.798-1.797) | 1.109 (0.667-1.844) | 1.116 (0.669-1.861) |
|  | 51-60 | 0.979 (0.833-1.150) | 0.979 (0.834-1.150) | 0.990 (0.839-1.168) | 0.993 (0.841-1.171) |
|  | >60 | 1.085 (0.971-1.212) | 1.085 (0.971-1.213) | 1.088 (0.969-1.222) | 1.090 (0.970-1.224) |

Abbreviations: PM_1_, particle with aerodynamic diameter ≤1.0 μm; PM_2.5,_ particle with aerodynamic diameter ≤2.5 μm; PM_10_, particle with aerodynamic diameter ≤10 μm; O_3_, ozone; NO_2,_ nitrogen dioxide.

^a^ Model I, unadjusted;

^b^ Model II, adjusted for sex;

^c^ Model III, adjusted for sex, educational level, residence (urban or rural), marital status, insurance, income group, cooking fuel, employment status, and sleep duration;

^d^ Model IV, adjusted for sex, educational level, residence (urban or rural), marital status, insurance, income group, cooking fuel, employment status, sleep duration, smoking status, and alcohol consumption;

* *P*<0.05; ** *P*<0.01; ****P*<0.001.

| **Supplementary Table S8.** The association between per 10 μg/m^3^ increase in PM_1_, PM_2.5_, PM_10_, O_3_, NO_2_ and incidence of cardiovascular diseases, and its major subtypes (heart disease and stroke) stratified by sex. | | | | | |
| --- | --- | --- | --- | --- | --- |
| Air pollutants exposure | Variable | Hazzard ratio (95% CI) |  |  |  |
|  |  | Model I ^a^ | Model II ^b^ | Model III ^c^ | Model IV ^d^ |
| Cardiovascular disease | | | | | |
| PM_1_ |  |  |  |  |  |
|  | Male | 1.130 (1.045-1.223) ** | 1.138 (1.051-1.233) ** | 1.136 (1.042-1.237) ** | 1.135 (1.042-1.237) ** |
|  | Female | 1.146 (1.070-1.228) *** | 1.152 (1.075-1.235) *** | 1.159 (1.077-1.248) *** | 1.164 (1.080-1.253) *** |
| PM_2.5_ |  |  |  |  |  |
|  | Male | 1.087 (1.044-1.133) *** | 1.092 (1.047-1.138) *** | 1.090 (1.042-1.140) *** | 1.090 (1.042-1.140) *** |
|  | Female | 1.096 (1.057-1.135) *** | 1.098 (1.059-1.138) *** | 1.100 (1.059-1.143) *** | 1.103 (1.062-1.146) *** |
| PM_10_ |  |  |  |  |  |
|  | Male | 1.069 (1.045-1.093) *** | 1.072 (1.049-1.097) *** | 1.069 (1.044-1.094) *** | 1.068 (1.044-1.093) *** |
|  | Female | 1.068 (1.048-1.088) *** | 1.070 (1.051-1.090) *** | 1.068 (1.047-1.089) *** | 1.069 (1.048-1.091) *** |
| O_3_ |  |  |  |  |  |
|  | Male | 1.010 (0.920-1.110) | 1.022 (0.931-1.121) | 1.011 (0.919-1.113) | 1.009 (0.917-1.111) |
|  | Female | 1.015 (0.937-1.099) | 1.021 (0.943-1.105) | 1.036 (0.954-1.126) | 1.036 (0.953-1.127) |
| NO_2_ |  |  |  |  |  |
|  | Male | 1.109 (1.025-1.200) * | 1.123 (1.036-1.217) ** | 1.122 (1.031-1.221) ** | 1.122 (1.030-1.221) ** |
|  | Female | 1.118 (1.043-1.199) ** | 1.123 (1.047-1.205) ** | 1.125 (1.045-1.212) ** | 1.129 (1.048-1.216) ** |
| Heart disease | | | | | |
| PM_1_ |  |  |  |  |  |
|  | Male | 1.134 (1.031-1.247) ** | 1.142 (1.037-1.259) ** | 1.138 (1.025-1.262) * | 1.139 (1.026-1.263) * |
|  | Female | 1.158 (1.071-1.252) *** | 1.162 (1.074-1.258) *** | 1.161 (1.068-1.261) *** | 1.165 (1.071-1.267) *** |
| PM_2.5_ |  |  |  |  |  |
|  | Male | 1.102 (1.049-1.159) *** | 1.107 (1.052-1.165) *** | 1.105 (1.047-1.168) *** | 1.106 (1.047-1.169) *** |
|  | Female | 1.110 (1.067-1.156) *** | 1.112 (1.068-1.158) *** | 1.112 (1.065-1.161) *** | 1.115 (1.067-1.164) *** |
| PM_10_ |  |  |  |  |  |
|  | Male | 1.082 (1.053-1.112) *** | 1.086 (1.057-1.116) *** | 1.082 (1.052-1.113) *** | 1.081 (1.051-1.112) *** |
|  | Female | 1.079 (1.057-1.101) *** | 1.081 (1.059-1.103) *** | 1.078 (1.055-1.101) *** | 1.079 (1.056-1.103) *** |
| O_3_ |  |  |  |  |  |
|  | Male | 1.067 (0.947-1.202) | 1.078 (0.957-1.214) | 1.084 (0.959-1.225) | 1.082 (0.957-1.223) |
|  | Female | 1.048 (0.956-1.148) | 1.053 (0.962-1.153) | 1.072 (0.974-1.179) | 1.072 (0.974-1.180) |
| NO_2_ |  |  |  |  |  |
|  | Male | 1.150 (1.044-1.266) ** | 1.163 (1.055-1.283) ** | 1.165 (1.051-1.291) ** | 1.165 (1.051-1.292) ** |
|  | Female | 1.135 (1.050-1.228) ** | 1.139 (1.053-1.232) ** | 1.139 (1.048-1.237) ** | 1.143 (1.051-1.242) ** |
| Stroke | | | | | |
| PM_1_ |  |  |  |  |  |
|  | Male | 1.120 (0.991-1.266) | 1.126 (0.993-1.277) | 1.128 (0.988-1.288) | 1.125 (0.985-1.285) |
|  | Female | 1.080 (0.957-1.219) | 1.083 (0.958-1.225) | 1.106 (0.971-1.259) | 1.113 (0.977-1.268) |
| PM_2.5_ |  |  |  |  |  |
|  | Male | 1.061 (0.997-1.130) | 1.064 (0.998-1.134) | 1.062 (0.992-1.136) | 1.060 (0.990-1.134) |
|  | Female | 1.040 (0.978-1.107) | 1.040 (0.977-1.108) | 1.047 (0.980-1.119) | 1.051 (0.983-1.124) |
| PM_10_ |  |  |  |  |  |
|  | Male | 1.037 (1.005-1.070) * | 1.040 (1.008-1.074) * | 1.037 (1.004-1.071) * | 1.037 (1.004-1.071) * |
|  | Female | 1.023 (0.990-1.057) | 1.024 (0.991-1.059) | 1.022 (0.988-1.058) | 1.024 (0.990-1.060) |
| O_3_ |  |  |  |  |  |
|  | Male | 0.973 (0.855-1.108) | 0.984 (0.865-1.119) | 0.947 (0.830-1.081) | 0.947 (0.829-1.080) |
|  | Female | 0.868 (0.755-0.998) * | 0.874 (0.762-1.004) | 0.879 (0.762-1.014) | 0.879 (0.761-1.015) |
| NO_2_ |  |  |  |  |  |
|  | Male | 1.050 (0.929-1.187) | 1.061 (0.936-1.203) | 1.060 (0.930-1.208) | 1.057 (0.928-1.205) |
|  | Female | 1.047 (0.923-1.188) | 1.050 (0.924-1.193) | 1.057 (0.926-1.207) | 1.063 (0.930-1.214) |

Abbreviations: PM_1_, particle with aerodynamic diameter ≤1.0 μm; PM_2.5,_ particle with aerodynamic diameter ≤2.5 μm; PM_10_, particle with aerodynamic diameter ≤10 μm; O_3_, ozone; NO_2,_ nitrogen dioxide.

^a^ Model I, unadjusted;

^b^ Model II, adjusted for age group;

^c^ Model III, adjusted for age group, educational level, residence (urban or rural), marital status, insurance, income group, cooking fuel, employment status, and sleep duration;

^d^ Model IV, adjusted for age group, educational level, residence (urban or rural), marital status, insurance, income group, cooking fuel, employment status, sleep duration, smoking status, and alcohol consumption;

* *P*<0.05; ** *P*<0.01; ****P*<0.001.

| **Supplementary Table S9.** The association between per 10 μg/m^3^ increase in PM_1_, PM_2.5_, PM_10_, O_3_, NO_2_ and incidence of cardiovascular diseases, and its major subtypes (heart disease and stroke) stratified by residence. | | | | | |
| --- | --- | --- | --- | --- | --- |
| Air pollutants exposure | Variable | Hazzard ratio (95% CI) |  |  |  |
|  |  | Model I ^a^ | Model II ^b^ | Model III ^c^ | Model IV ^d^ |
| Cardiovascular disease | | | | | |
| PM_1_ |  |  |  |  |  |
|  | Rural  Urban | 1.164 (1.093-1.239) *** | 1.175 (1.102-1.253) *** | 1.187 (1.109-1.270) *** | 1.188 (1.110-1.271) *** |
|  |  | 1.093 (0.998-1.197) | 1.092 (0.995-1.198) | 1.069 (0.969-1.180) | 1.069 (0.968-1.179) |
| PM_2.5_ |  |  |  |  |  |
|  | Rural | 1.102 (1.067-1.138) *** | 1.107 (1.072-1.144) *** | 1.111 (1.074-1.151) *** | 1.112 (1.074-1.152) *** |
|  | Urban | 1.073 (1.022-1.126) ** | 1.072 (1.020-1.126) ** | 1.060 (1.005-1.117) * | 1.060 (1.005-1.117) * |
| PM_10_ |  |  |  |  |  |
|  | Rural | 1.075 (1.056-1.094) *** | 1.080 (1.061-1.099) *** | 1.080 (1.060-1.100) *** | 1.080 (1.060-1.100) *** |
|  | Urban | 1.058 (1.032-1.084) *** | 1.058 (1.033-1.085) *** | 1.050 (1.022-1.077) *** | 1.050 (1.023-1.078) *** |
| O_3_ |  |  |  |  |  |
|  | Rural | 1.017 (0.940-1.100) | 1.032 (0.955-1.116) | 1.043 (0.962-1.130) | 1.040 (0.959-1.128) |
|  | Urban | 1.002 (0.910-1.103) | 0.995 (0.904-1.096) | 0.997 (0.902-1.102) | 0.997 (0.902-1.103) |
| NO_2_ |  |  |  |  |  |
|  | Rural | 1.156 (1.085-1.232) *** | 1.172 (1.100-1.250) *** | 1.179 (1.102-1.262) *** | 1.179 (1.101-1.262) *** |
|  | Urban | 1.031 (0.940-1.131) | 1.029 (0.937-1.130) | 1.024 (0.930-1.128) | 1.025 (0.931-1.129) |
| Heart disease | | | | | |
| PM_1_ |  |  |  |  |  |
|  | Rural | 1.176 (1.093-1.265) *** | 1.187 (1.102-1.279) *** | 1.200 (1.109-1.298) *** | 1.200 (1.110-1.299) *** |
|  | Urban | 1.096 (0.984-1.221) | 1.095 (0.981-1.221) | 1.061 (0.946-1.191) | 1.061 (0.945-1.191) |
| PM_2.5_ |  |  |  |  |  |
|  | Rural | 1.113 (1.073-1.156) *** | 1.119 (1.077-1.162) *** | 1.124 (1.080-1.171) *** | 1.125 (1.081-1.171) *** |
|  | Urban | 1.096 (1.035-1.161) ** | 1.095 (1.034-1.161) ** | 1.078 (1.014-1.147) * | 1.078 (1.013-1.147) * |
| PM_10_ |  |  |  |  |  |
|  | Rural | 1.083 (1.061-1.105) *** | 1.087 (1.066-1.110) *** | 1.088 (1.065-1.112) *** | 1.088 (1.065-1.112) *** |
|  | Urban | 1.076 (1.047-1.107) *** | 1.077 (1.047-1.085) *** | 1.066 (1.035-1.099) *** | 1.067 (1.035-1.100) *** |
| O_3_ |  |  |  |  |  |
|  | Rural | 1.035 (0.941-1.138) | 1.049 (0.955-1.152) | 1.074 (0.975-1.183) | 1.072 (0.972-1.181) |
|  | Urban | 1.085 (0.969-1.214) | 1.077 (0.962-1.206) | 1.081 (0.960-1.217) | 1.082 (0.961-1.219) |
| NO_2_ |  |  |  |  |  |
|  | Rural | 1.186 (1.103-1.276) *** | 1.202 (1.116-1.294) *** | 1.218 (1.126-1.317) *** | 1.217 (1.125-1.316) *** |
|  | Urban | 1.052 (0.943-1.173) | 1.048 (0.939-1.170) | 1.037 (0.927-1.161) | 1.040 (0.928-1.164) |
| Stroke | | | | | |
| PM_1_ |  |  |  |  |  |
|  | Rural | 1.109 (0.996-1.235) | 1.121 (1.004-1.250) * | 1.134 (1.011-1.272) * | 1.136 (1.013-1.275) * |
|  | Urban | 1.084 (0.938-1.253) | 1.078 (0.929-1.251) | 1.074 (0.917-1.257) | 1.075 (0.918-1.258) |
| PM_2.5_ |  |  |  |  |  |
|  | Rural | 1.064 (1.008-1.123) * | 1.069 (1.012-1.129) * | 1.072 (1.012-1.136) * | 1.073 (1.013-1.137) * |
|  | Urban | 1.024 (0.948-1.107) | 1.021 (0.943-1.105) | 1.017 (0.934-1.106) | 1.018 (0.935-1.107) |
| PM_10_ |  |  |  |  |  |
|  | Rural | 1.045 (1.015-1.075) ** | 1.049 (1.019-1.080) ** | 1.048 (1.017-1.081) ** | 1.049 (1.017-1.081) ** |
|  | Urban | 1.004 (0.967-1.043) | 1.003 (0.965-1.042) | 0.998 (0.959-1.039) | 0.999 (0.960-1.040) |
| O_3_ |  |  |  |  |  |
|  | Rural | 0.941 (0.833-1.063) | 0.960 (0.852-1.082) | 0.948 (0.838-1.073) | 0.944 (0.834-1.068) |
|  | Urban | 0.876 (0.749-1.024) | 0.866 (0.739-1.015) | 0.859 (0.731-1.009) | 0.860 (0.731-1.012) |
| NO_2_ |  |  |  |  |  |
|  | Rural | 1.053 (0.944-1.174) | 1.068 (0.955-1.193) | 1.060 (0.944-1.191) | 1.060 (0.944-1.190) |
|  | Urban | 1.040 (0.897-1.207) | 1.034 (0.889-1.203) | 1.038 (0.888-1.215) | 1.040 (0.888-1.217) |

Abbreviations: PM_1_, particle with aerodynamic diameter ≤1.0 μm; PM_2.5,_ particle with aerodynamic diameter ≤2.5 μm; PM_10_, particle with aerodynamic diameter ≤10 μm; O_3_, ozone; NO_2,_ nitrogen dioxide.

^a^ Model I, unadjusted;

^b^ Model II, adjusted for age group and sex;

^c^ Model III, adjusted for age group, sex, educational level, marital status, insurance, income group, cooking fuel, employment status, and sleep duration;

^d^ Model IV, adjusted for age group, sex, educational level, marital status, insurance, income group, cooking fuel, employment status, sleep duration, smoking status, and alcohol consumption;

* *P*<0.05; ** *P*<0.01; ****P*<0.001.

| **Supplementary Table S10.** The association between per 10 μg/m^3^ increase in PM_1_, PM_2.5_, PM_10_, O_3_, NO_2_ and incidence of cardiovascular diseases, and its major subtypes (heart disease and stroke) stratified by education level. | | | | | |
| --- | --- | --- | --- | --- | --- |
| Air pollutants exposure | Variable | Hazzard ratio (95% CI) |  |  |  |
|  |  | Model I ^a^ | Model II ^b^ | Model III ^c^ | Model IV ^d^ |
| Cardiovascular disease | | | | | |
| PM_1_ |  |  |  |  |  |
|  | Elementary school or below  Middle school and high school  Technical school and above | 1.207 (1.133-1.286) *** | 1.197 (1.123-1.276) *** | 1.209 (1.129-1.294) *** | 1.210 (1.131-1.295) *** |
|  |  | 1.035 (0.936-1.144) | 1.036 (0.936-1.146) | 1.049 (0.945-1.165) | 1.048 (0.944-1.164) |
|  |  | 0.927 (0.708-1.214) | 0.904 (0.683-1.196) | 0.918 (0.692-1.216) | 0.884 (0.660-1.184) |
| PM_2.5_ |  |  |  |  |  |
|  | Elementary school or below | 1.124 (1.088-1.160) *** | 1.118 (1.082-1.155) *** | 1.122 (1.084-1.162) *** | 1.123 (1.085-1.163) *** |
|  | Middle school and high school | 1.041 (0.987-1.097) | 1.041 (0.987-1.099) | 1.049 (0.992-1.110) | 1.049 (0.992-1.110) |
|  | Technical school and above | 0.971 (0.841-1.121) | 0.963 (0.830-1.117) | 0.959 (0.824-1.116) | 0.952 (0.815-1.112) |
| PM_10_ |  |  |  |  |  |
|  | Elementary school or below | 1.084 (1.066-1.102) *** | 1.083 (1.065-1.102) *** | 1.082 (1.063-1.101) *** | 1.082 (1.063-1.101) *** |
|  | Middle school and high school | 1.044 (1.013-1.075) ** | 1.044 (1.013-1.075) ** | 1.047 (1.016-1.080) ** | 1.047 (1.016-1.079) ** |
|  | Technical school and above | 1.000 (0.928-1.077) | 1.000 (0.927-1.079) | 0.991 (0.920-1.068) | 1.003 (0.924-1.087) |
| O_3_ |  |  |  |  |  |
|  | Elementary school or below | 1.020 (0.948-1.098) | 1.019 (0.948-1.096) | 1.038 (0.962-1.119) | 1.035 (0.960-1.117) |
|  | Middle school and high school | 1.023 (0.911-1.150) | 1.029 (0.915-1.157) | 1.020 (0.906-1.149) | 1.021 (0.906-1.150) |
|  | Technical school and above | 0.871 (0.644-1.177) | 0.867 (0.640-1.174) | 0.832 (0.611-1.134) | 0.877 (0.643-1.195) |
| NO_2_ |  |  |  |  |  |
|  | Elementary school or below | 1.179 (1.107-1.256) *** | 1.172 (1.099-1.250) *** | 1.185 (1.107-1.267) *** | 1.184 (1.107-1.267) *** |
|  | Middle school and high school | 1.021 (0.923-1.130) | 1.024 (0.925-1.135) | 1.033 (0.930-1.148) | 1.033 (0.930-1.148) |
|  | Technical school and above | 0.859 (0.660-1.117) | 0.842 (0.649-1.092) | 0.854 (0.652-1.118) | 0.852 (0.647-1.122) |
| Heart disease | | | | | |
| PM_1_ |  |  |  |  |  |
|  | Elementary school or below | 1.219 (1.132-1.313) *** | 1.206 (1.119-1.300) *** | 1.224 (1.130-1.325) *** | 1.225 (1.131-1.326) *** |
|  | Middle school and high school | 1.041 (0.927-1.168) | 1.039 (0.925-1.168) | 1.046 (0.927-1.180) | 1.046 (0.927-1.179) |
|  | Technical school and above | 0.900 (0.658-1.231) | 0.889 (0.641-1.232) | 0.920 (0.667-1.268) | 0.891 (0.640-1.239) |
| PM_2.5_ |  |  |  |  |  |
|  | Elementary school or below | 1.141 (1.099-1.185) *** | 1.134 (1.092-1.179) *** | 1.143 (1.097-1.190) *** | 1.143 (1.098-1.191) *** |
|  | Middle school and high school | 1.052 (0.989-1.119) | 1.052 (0.988-1.120) | 1.057 (0.991-1.128) | 1.058 (0.992-1.128) |
|  | Technical school and above | 0.966 (0.819-1.141) | 0.967 (0.814-1.150) | 0.973 (0.821-1.154) | 0.966 (0.812-1.150) |
| PM_10_ |  |  |  |  |  |
|  | Elementary school or below | 1.096 (1.076-1.117) *** | 1.095 (1.074-1.116) *** | 1.096 (1.074-1.118) *** | 1.096 (1.074-1.118) *** |
|  | Middle school and high school | 1.054 (1.017-1.091) ** | 1.052 (1.017-1.089) ** | 1.055 (1.019-1.093) ** | 1.055 (1.019-1.093) ** |
|  | Technical school and above | 1.009 (0.927-1.098) | 1.014 (0.930-1.106) | 1.007 (0.928-1.093) | 1.017 (0.930-1.111) |
| O_3_ |  |  |  |  |  |
|  | Elementary school or below | 1.056 (0.966-1.153) | 1.051 (0.963-1.147) | 1.084 (0.989-1.187) | 1.081 (0.986-1.185) |
|  | Middle school and high school | 1.095 (0.957-1.253) | 1.099 (0.959-1.259) | 1.103 (0.962-1.264) | 1.104 (0.963-1.266) |
|  | Technical school and above | 0.860 (0.589-1.256) | 0.868 (0.593-1.271) | 0.797 (0.537-1.182) | 0.831 (0.559-1.235) |
| NO_2_ |  |  |  |  |  |
|  | Elementary school or below | 1.203 (1.117-1.295) *** | 1.193 (1.108-1.286) *** | 1.219 (1.127-1.318) *** | 1.218 (1.126-1.318) *** |
|  | Middle school and high school | 1.053 (0.937-1.184) | 1.052 (0.935-1.183) | 1.053 (0.934-1.188) | 1.054 (0.935-1.189) |
|  | Technical school and above | 0.880 (0.650-1.190) | 0.871 (0.645-1.177) | 0.903 (0.670-1.217) | 0.904 (0.667-1.225) |
| Stroke | | | | | |
| PM_1_ |  |  |  |  |  |
|  | Elementary school or below | 1.156 (1.041-1.282) ** | 1.147 (1.031-1.276) * | 1.151 (1.030-1.287) * | 1.154 (1.033-1.290) * |
|  | Middle school and high school | 0.994 (0.838-1.180) | 1.000 (0.842-1.189) | 1.043 (0.870-1.252) | 1.043 (0.869-1.252) |
|  | Technical school and above | 1.146 (0.746-1.761) | 1.075 (0.676-1.710) | 0.948 (0.560-1.595) | 0.943 (0.570-1.561) |
| PM_2.5_ |  |  |  |  |  |
|  | Elementary school or below | 1.080 (1.024-1.138) ** | 1.074 (1.018-1.134) ** | 1.074 (1.015-1.136) * | 1.075 (1.016-1.138) * |
|  | Middle school and high school | 0.991 (0.906-1.083) | 0.994 (0.909-1.088) | 1.014 (0.922-1.116) | 1.014 (0.921-1.115) |
|  | Technical school and above | 1.057 (0.832-1.342) | 1.023 (0.794-1.317) | 0.941 (0.707-1.253) | 0.954 (0.725-1.255) |
| PM_10_ |  |  |  |  |  |
|  | Elementary school or below | 1.046 (1.018-1.074) *** | 1.045 (1.017-1.073) ** | 1.040 (1.012-1.069) ** | 1.041 (1.013-1.070) ** |
|  | Middle school and high school | 1.000 (0.954-1.049) | 1.003 (0.956-1.052) | 1.011 (0.963-1.062) | 1.011 (0.962-1.062) |
|  | Technical school and above | 1.006 (0.902-1.123) | 0.993 (0.882-1.118) | 0.964 (0.845-1.101) | 0.979 (0.855-1.122) |
| O_3_ |  |  |  |  |  |
|  | Elementary school or below | 0.925 (0.828-1.034) | 0.931 (0.834-1.039) | 0.921 (0.821-1.032) | 0.920 (0.820-1.031) |
|  | Middle school and high school | 0.908 (0.744-1.110) | 0.917 (0.751-1.120) | 0.902 (0.735-1.109) | 0.900 (0.733-1.106) |
|  | Technical school and above | 0.983 (0.644-1.500) | 0.927 (0.608-1.412) | 0.889 (0.567-1.395) | 0.942 (0.566-1.567) |
| NO_2_ |  |  |  |  |  |
|  | Elementary school or below | 1.100 (0.990-1.223) | 1.095 (0.983-1.220) | 1.087 (0.972-1.215) | 1.089 (0.974-1.217) |
|  | Middle school and high school | 0.947 (0.797-1.125) | 0.958 (0.805-1.139) | 1.006 (0.838-1.208) | 1.005 (0.837-1.207) |
|  | Technical school and above | 1.066 (0.661-1.720) | 1.004 (0.616-1.639) | 0.846 (0.484-1.480) | 0.835 (0.478-1.457) |

Abbreviations: PM_1_, particle with aerodynamic diameter ≤1.0 μm; PM_2.5,_ particle with aerodynamic diameter ≤2.5 μm; PM_10_, particle with aerodynamic diameter ≤10 μm; O_3_, ozone; NO_2,_ nitrogen dioxide.

^a^ Model I, unadjusted;

^b^ Model II, adjusted for age group and sex;

^c^ Model III, adjusted for age group, sex, residence (urban or rural), marital status, insurance, income group, cooking fuel, employment status, and sleep duration;

^d^ Model IV, adjusted for age group, sex, residence (urban or rural), marital status, insurance, income group, cooking fuel, employment status, sleep duration, smoking status, and alcohol consumption;

* *P*<0.05; ** *P*<0.01; ****P*<0.001.

| **Supplementary Table S11**. The associations between per 10 μg/m^3^ increase in PM_1_, PM_2.5_, PM_10_, O_3_, NO_2_ and incidence of cardiovascular diseases across cardiovascular-kidney-metabolic (CKM) syndrome stages: the results of time-varying cox regression analysis. | | | | |
| --- | --- | --- | --- | --- |
| Air pollutants exposure | Hazzard ratio (95% CI) |  |  |  |
|  | Model I ^a^ | Model II ^b^ | Model III ^c^ | Model IV ^d^ |
| CKM stage 0-1 | | | | |
| PM_1_ | 1.190 (1.045-1.354) ** | 1.195 (1.047-1.365) ** | 1.207 (1.047-1.391) ** | 1.210 (1.050-1.395) ** |
| PM_2.5_ | 1.114 (1.042-1.192) ** | 1.116 (1.042-1.195) ** | 1.120 (1.041-1.206) ** | 1.122 (1.042-1.208) ** |
| PM_10_ | 1.076 (1.038-1.117) *** | 1.077 (1.039-1.117) *** | 1.076 (1.036-1.118) *** | 1.077 (1.037-1.118) *** |
| O_3_ | 1.056 (0.920-1.211) | 1.067 (0.930-1.222) | 1.108 (0.965-1.272) | 1.108 (0.965-1.272) |
| NO_2_ | 1.126 (0.984-1.288) | 1.137 (0.992-1.304) | 1.176 (1.019-1.357) * | 1.178 (1.020-1.360) * |
| CKM stage 2-3 | | | | |
| PM_1_ | 1.126 (1.064-1.190) *** | 1.133 (1.070-1.199) *** | 1.130 (1.064-1.200) *** | 1.131 (1.065-1.201) *** |
| PM_2.5_ | 1.085 (1.054-1.117) *** | 1.088 (1.057-1.121) *** | 1.087 (1.053-1.121) *** | 1.087 (1.053-1.122) *** |
| PM_10_ | 1.064 (1.048-1.081) *** | 1.068 (1.052-1.085) *** | 1.065 (1.047-1.082) *** | 1.065 (1.047-1.082) *** |
| O_3_ | 1.001 (0.936-1.071) | 1.007 (0.942-1.077) | 1.005 (0.937-1.078) | 1.004 (0.936-1.077) |
| NO_2_ | 1.103 (1.042-1.168) *** | 1.112 (1.050-1.178) *** | 1.106 (1.041-1.175) ** | 1.106 (1.041-1.175) ** |

Abbreviations: PM_1_, particle with aerodynamic diameter ≤1.0 μm; PM_2.5,_ particle with aerodynamic diameter ≤2.5 μm; PM_10_, particle with aerodynamic diameter ≤10 μm; O_3_, ozone; NO_2,_ nitrogen dioxide.

^a^ Model I, unadjusted;

^b^ Model II, adjusted for age group and sex;

^c^ Model III, adjusted for age group, sex, educational level, residence (urban or rural), marital status, insurance, income group, cooking fuel, employment status, and sleep duration;

^d^ Model IV, adjusted for age group, sex, educational level, residence (urban or rural), marital status, insurance, income group, cooking fuel, employment status, sleep duration, smoking status, and alcohol consumption.

* *P*<0.05; ** *P*<0.01; ****P*<0.001.

| **Supplementary Table S12.** The associations between per 10 μg/m^3^ increase in PM_1_, PM_2.5_, PM_10_, O_3_, NO_2_ and incidence of cardiovascular diseases in 6,962 cardiovascular-kidney-metabolic (CKM) syndrome stages 0-3 participants: the results of sensitivity analysis where participants developing cardiovascular diseases in the first two follow-up waves were excluded. | | | | |
| --- | --- | --- | --- | --- |
| Air pollutants exposure | Hazzard ratio (95% CI) |  |  |  |
|  | Model I ^a^ | Model II ^b^ | Model III ^c^ | Model IV ^d^ |
| PM_1_ | 1.193 (1.115-1.277) *** | 1.201 (1.121-1.287) *** | 1.202 (1.117-1.292) *** | 1.203 (1.119-1.294) *** |
| PM_2.5_ | 1.114 (1.077-1.153) *** | 1.118 (1.079-1.157) *** | 1.118 (1.077-1.160) *** | 1.118 (1.078-1.160) *** |
| PM_10_ | 1.075 (1.055-1.095) *** | 1.078 (1.058-1.098) *** | 1.076 (1.055-1.096) *** | 1.076 (1.056-1.097) *** |
| O_3_ | 1.005 (0.936-1.079) | 1.009 (0.940-1.083) | 1.017 (0.946-1.093) | 1.018 (0.946-1.094) |
| NO_2_ | 1.108 (1.033-1.190) ** | 1.117 (1.039-1.200) ** | 1.119 (1.038-1.206) ** | 1.121 (1.040-1.208) ** |

Abbreviations: PM_1_, particle with aerodynamic diameter ≤1.0 μm; PM_2.5,_ particle with aerodynamic diameter ≤2.5 μm; PM_10_, particle with aerodynamic diameter ≤10 μm; O_3_, ozone; NO_2,_ nitrogen dioxide.

^a^ Model I, unadjusted;

^b^ Model II, adjusted for age group and sex;

^c^ Model III, adjusted for age group, sex, educational level, residence (urban or rural), marital status, insurance, income group, cooking fuel, employment status, and sleep duration;

^d^ Model IV, adjusted for age group, sex, educational level, residence (urban or rural), marital status, insurance, income group, cooking fuel, employment status, sleep duration, smoking status, and alcohol consumption.

* *P*<0.05; ** *P*<0.01; *** *P*<0.001.

| **Supplementary Table S13**. The associations between per 10 μg/m^3^ increase in PM_1_, PM_2.5_, PM_10_, O_3_, NO_2_ and incidence of cardiovascular diseases in 7,400 cardiovascular-kidney-metabolic (CKM) syndrome stages 0-3 participants: the results of time-varying cox regression analysis (lag effects were adjusted for 2-years). | | | | |
| --- | --- | --- | --- | --- |
| Air pollutants exposure | Hazzard ratio (95% CI) |  |  |  |
|  | Model I ^a^ | Model II ^b^ | Model III ^c^ | Model IV ^d^ |
| PM_1_ | 1.112 (1.060-1.166) *** | 1.117 (1.064-1.172) *** | 1.115 (1.059-1.174) *** | 1.116 (1.060-1.175) *** |
| PM_2.5_ | 1.076 (1.050-1.103) *** | 1.078 (1.051-1.106) *** | 1.077 (1.048-1.106) *** | 1.078 (1.049-1.107) *** |
| PM_10_ | 1.060 (1.046-1.074) *** | 1.063 (1.048-1.077) *** | 1.060 (1.045-1.075) *** | 1.060 (1.045-1.076) *** |
| O_3_ | 0.910 (0.838-0.988) * | 0.909 (0.837-0.988) * | 0.915 (0.840-0.996) * | 0.914 (0.839-0.996) * |
| NO_2_ | 1.108 (1.055-1.164) *** | 1.116 (1.062-1.173) *** | 1.115 (1.058-1.175) *** | 1.115 (1.058-1.175) *** |

Abbreviations: PM_1_, particle with aerodynamic diameter ≤1.0 μm; PM_2.5,_ particle with aerodynamic diameter ≤2.5 μm; PM_10_, particle with aerodynamic diameter ≤10 μm; O_3_, ozone; NO_2,_ nitrogen dioxide.

^a^ Model I, unadjusted;

^b^ Model II, adjusted for age group and sex;

^c^ Model III, adjusted for age group, sex, educational level, residence (urban or rural), marital status, insurance, income group, cooking fuel, employment status, and sleep duration;

^d^ Model IV, adjusted for age group, sex, educational level, residence (urban or rural), marital status, insurance, income group, cooking fuel, employment status, sleep duration, smoking status, and alcohol consumption.

* *P*<0.05; ** *P*<0.01; ****P*<0.001.

# **Supplementary Figure S1.** City-level annual concentration exposure distribution maps of air pollutants for PM_1_, PM_2.5_, PM_10_, O_3_, and NO_2_ in China.


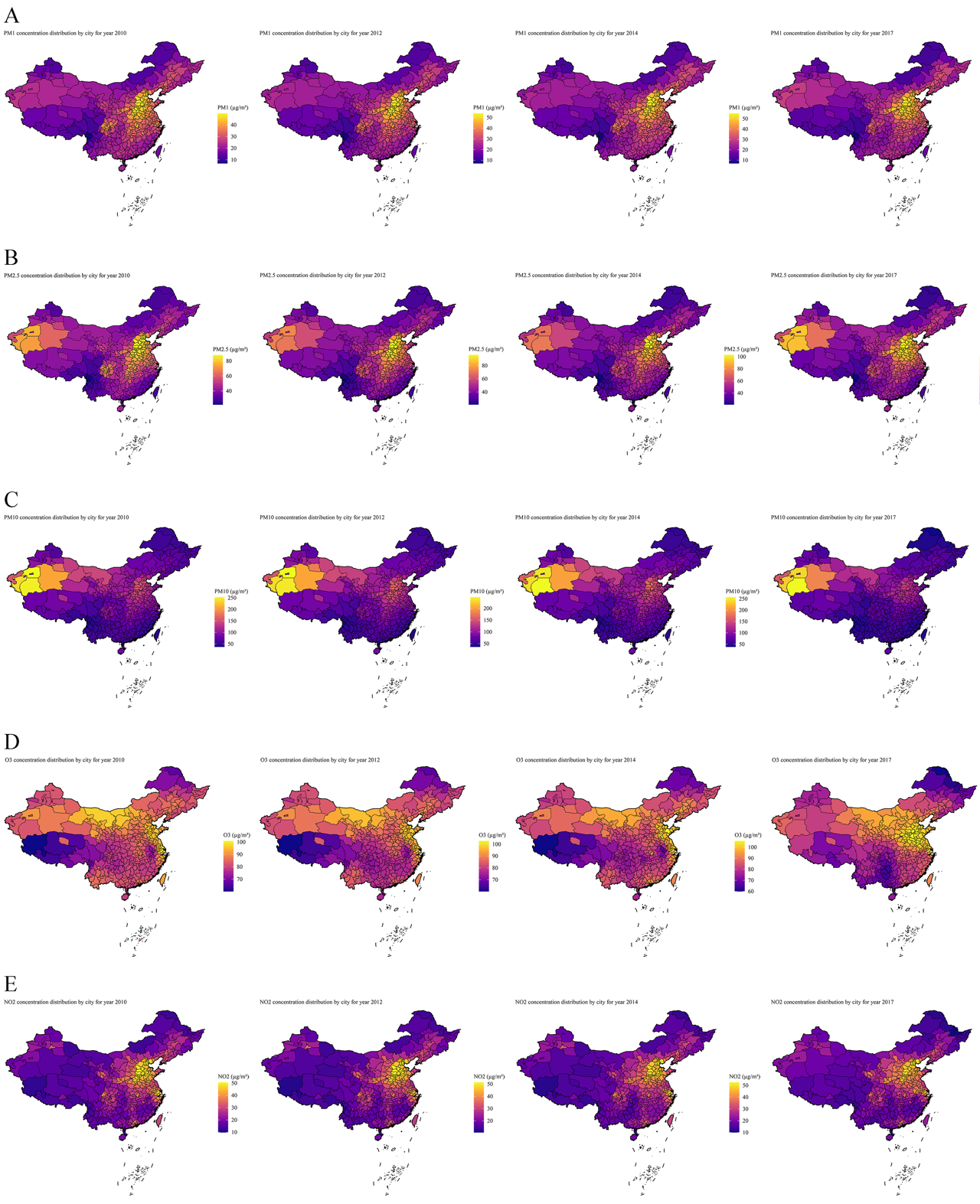


Note: (A) particle with aerodynamic diameter ≤1.0 μm (PM_1)_ map for the years 2010, 2012, 2015, 2017 and average; (B) particle with aerodynamic diameter ≤2.5 μm (PM_2.5)_ map for the years 2010, 2012, 2015, 2017 and average; (C) particle with aerodynamic diameter ≤10 μm (PM_10)_ map for the years 2010, 2012, 2015, 2017 and average; (D) ozone (O_3)_ map for the years 2010, 2012, 2015, 2017 and average; (E) nitrogen dioxide (NO_2)_ map for the years 2010, 2012, 2015, 2017 and average.

# **Supplementary Figure S2.** Average annual concentration of air pollutants in PM_1_, PM_2.5_, PM_10_, O_3_, and NO_2_ by cardiovascular disease status (median, interquartile range) during their follow-up.


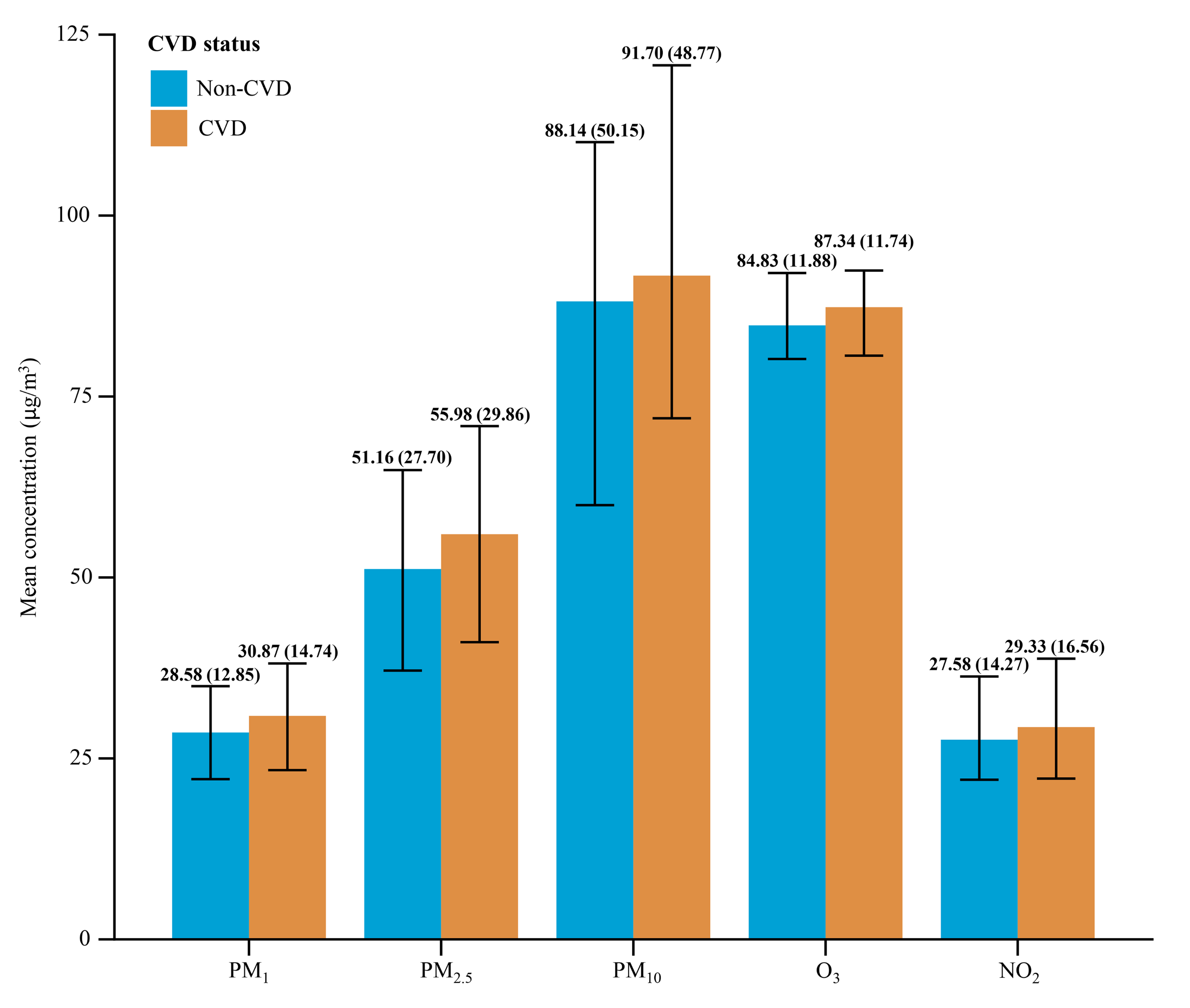


Note: PM_1_, particle with aerodynamic diameter ≤1.0 μm; PM_2.5,_ particle with aerodynamic diameter ≤2.5 μm; PM_10_, particle with aerodynamic diameter ≤10 μm; O_3_, ozone; NO_2,_ nitrogen dioxide; CVD, cardiovascular disease.

**Reference**

1. Ndumele CE, Rangaswami J, Chow SL, Neeland IJ, Tuttle KR, Khan SS, Coresh J, Mathew RO, Baker-Smith CM, Carnethon MR, et al. Cardiovascular-kidney-metabolic health: a presidential advisory from the american heart association. *Circulation* (2023) 148:1606–1635. doi: 10.1161/CIR.0000000000001184

2. Inker LA, Eneanya ND, Coresh J, Tighiouart H, Wang D, Sang Y, Crews DC, Doria A, Estrella MM, Froissart M, et al. New creatinine- and cystatin C-based equations to estimate GFR without race. *N Engl J Med* (2021) 385:1737–1749. doi: 10.1056/NEJMoa2102953

3. Khan SS, Matsushita K, Sang Y, Ballew SH, Grams ME, Surapaneni A, Blaha MJ, Carson AP, Chang AR, Ciemins E, et al. Development and validation of the american heart association’s PREVENT equations. *Circulation* (2024) 149:430–449. doi: 10.1161/CIRCULATIONAHA.123.067626

4. Aggarwal R, Ostrominski JW, Vaduganathan M. Prevalence of cardiovascular-kidney-metabolic syndrome stages in US adults, 2011-2020. *JAMA* (2024) 331:1858–1860. doi: 10.1001/jama.2024.6892

5. WHO global air quality guidelines: particulate matter (PM2.5 and PM10), ozone, nitrogen dioxide, sulfur dioxide and carbon monoxide. Geneva: World Health Organization. (2021). http://www.ncbi.nlm.nih.gov/books/NBK574594/ [Accessed July 13, 2025]
